# Faecalibacterium prausnitzii as a potential Antiatherosclerotic microbe

**DOI:** 10.1186/s12964-023-01464-y

**Published:** 2024-01-19

**Authors:** Hai-Tao Yang, Zhi-hui Jiang, Yi Yang, Ting-Ting Wu, Ying-Ying Zheng, Yi-Tong Ma, Xiang Xie

**Affiliations:** Department of Cardiology, First Affiliated Hospital of Xinjiang Medical University, No.137, Liyushan Road, Urumqi, 830011 China

**Keywords:** Coronary artery disease, *Faecalibacterium prausnitzii*, Gut microbiota, Lipopolysaccharide

## Abstract

**Background:**

The gut microbiota plays a crucial role in coronary artery disease (CAD) development, but limited attention has been given to the role of the microbiota in preventing this disease. This study aimed to identify key biomarkers using metagenomics and untargeted metabolomics and verify their associations with atherosclerosis.

**Methods:**

A total of 371 participants, including individuals with various CAD types and CAD-free controls, were enrolled. Subsequently, significant markers were identified in the stool samples through gut metagenomic sequencing and untargeted metabolomics. In vivo and in vitro experiments were performed to investigate the mechanisms underlying the association between these markers and atherosclerosis.

**Results:**

Faecal omics sequencing revealed that individuals with a substantial presence of *Faecalibacterium prausnitzii* had the lowest incidence of CAD across diverse CAD groups and control subjects. A random forest model confirmed the significant relationship between *F. prausnitzii* and CAD incidence. Notably, *F. prausnitzii* emerged as a robust, independent CAD predictor. Furthermore, our findings indicated the potential of the gut microbiota and gut metabolites to predict CAD occurrence and progression, potentially impacting amino acid and vitamin metabolism. *F. prausnitzii* mitigated inflammation and exhibited an antiatherosclerotic effect on ApoE^−/−^ mice after gavage. This effect was attributed to reduced intestinal LPS synthesis and reinforced mechanical and mucosal barriers, leading to decreased plasma LPS levels and an antiatherosclerotic outcome.

**Conclusions:**

Sequencing of the samples revealed a previously unknown link between specific gut microbiota and atherosclerosis. Treatment with *F. prausnitzii* may help prevent CAD by inhibiting atherosclerosis.

**Supplementary Information:**

The online version contains supplementary material available at 10.1186/s12964-023-01464-y.

## Introduction

The prevalence of coronary artery disease (CAD) is on the rise due to the ageing of the population. In 2020, CAD affected approximately 11.39 million individuals in China alone, and its mortality rate remains high [1]. Despite advancements in diagnostic tools and treatment modalities, CAD continues to exact a high toll in terms of mortality. According to a cross-sectional study of 12,695 patients, the 1-year mortality rate was 28% in patients with acute myocardial infarction and 32.1% in patients with recurrent myocardial infarction [2]. It is very important to find new therapeutic directions and explore new therapeutic targets for the diagnosis and treatment of CAD.

With the continuous improvement of sequencing technology, the gut microbiota has been gradually found to play a key role in the occurrence and development of CAD. Among the emerging probiotics, *Faecalibacterium prausnitzii* has garnered substantial attention. *F. prausnitzii*, abundant in the human intestinal microbiota, is known for its production of butyrate [3]. Notably, previous research has suggested that *F. prausnitzii* may have beneficial effects in the context of renal failure, attributed to its butyrate production [4]. Recent investigations into the composition of the intestinal microbiota in individuals with various health conditions, particularly CAD, have revealed a notable decrease in the abundance of *F. prausnitzii* [5–9]. Given that the development of CAD is closely associated with the progression of atherosclerotic plaques, intriguing questions about whether *F. prausnitzii* plays a pivotal role in inhibiting atherosclerotic plaque formation should be raised. Furthermore, it is currently unclear whether *F. prausnitzii* enhances vascular health through its production of butyrate, thereby potentially contributing to the prevention of atherosclerotic plaque formation. The mechanisms underlying these potential effects of *F. prausnitzii* are yet to be fully understood. However, further research is needed to clarify the complex relationship between *F. prausnitzii* and atherosclerosis. CAD continues to be a prominent global cause of morbidity and mortality, despite the widespread use of statin therapy [10, 11]. Our study aimed to pinpoint specific elements of the gut microbiota that could serve as promising targets for innovative and cost-effective strategies to prevent CAD. We collected faecal samples from various clinical groups, including individuals with acute myocardial infarction, unstable angina pectoris, and stable angina pectoris and those without CAD. These samples were subjected to simultaneous metagenomic sequencing and untargeted metabolomics analysis. Through bioinformatics analysis, we identified key gut microbiota components that could be useful for preventing CAD. Furthermore, we conducted both in vivo and in vitro experiments to elucidate the mechanisms underlying the association between *F. prausnitzii* and atherosclerosis.

## Methods

### Study design and participant selection

A total of 215 patients diagnosed with CAD were recruited from December 2019 to June 2020 at the First Affiliated Hospital of Xinjiang Medical University. This cohort included 56 patients with stable coronary artery disease (SCAD), 106 patients with unstable angina (UA), 53 patients with myocardial infarction (MI), and 156 control subjects without CAD. CAD diagnosis was confirmed through coronary angiography, with CAD defined as at least one coronary artery stenosis ≥50%. The controls were individuals without CAD who had risk factors for CAD and who underwent either coronary CT or coronary angiography with negative results. The detailed diagnostic criteria for the CAD patients, including the exclusion criteria, are summarized in the Supplementary Methods.

In this study, metagenomic sequencing was performed on the faeces of each participant, involving a total of 371 individuals. Subsequently, a validation cohort consisting of 50 CAD patients and 50 healthy controls was established; the patients were randomly selected from the overall population. This subgroup included 15 SCAD patients, 28 UA patients, and 7 MI patients. Faecal untargeted metabolomics was conducted in the validation cohort for further analysis. All participants provided written informed consent at the time of study registration, and the study design complied with the Declaration of Helsinki. This study was approved by the Ethics Committee of the First Affiliated Hospital of Xinjiang Medical University (approval no. 20190905–16) and registered in the Chinese Clinical Trial Registry Network (trial registration no. ChiCTR1900027476).

### Participant characteristics and data collection

Considering alcohol intake, subjects who drank more than 30 g of alcohol per day were classified as heavy drinkers [12, 13]. For smoking, smokers who had smoked continuously or for an accumulated total of 6 months or more were defined as current smokers. Smokers who had stopped smoking at the time of the survey and persisted for more than 6 months were referred to as past smokers [12]. The inclusion criteria for diabetic patients were as follows: 1. had a fasting blood glucose level ≥ 7.0 mmol/L at admission and/or 2-hour postprandial blood glucose ≥11.1 mmol/L [14]; and 2. had previously been diagnosed with diabetes and used antidiabetic drugs. The inclusion criteria for hypertensive patients were as follows: 1. no antihypertensive drugs, ≥140 mmHg systolic blood pressure and/or ≥ 90 mmHg diastolic blood pressure measured at least three times on the same day [15]; and 2. previously diagnosed with hypertension and taking oral antihypertensive drugs.

Five millilitres of peripheral venous blood was collected from each patient after 12 hours of fasting. The laboratory data included liver function parameters, renal function parameters, blood lipids and blood glucose levels. A disposable sterile bag was used for each participant to collect faeces, and a stool sampler with a spoon (X517, Xinkang, Jiangsu, China) was used for sample collection. All participants were trained to collect samples and were instructed to empty their urine before retaining stool, wash their hands adequately and wear disposable gloves before collecting the stool samples. Stool samples obtained from each participant were shipped to the laboratory within 2 hours and frozen at − 80 °C.

### Laboratory analyses and metagenomic sequencing

All analyses were performed by Shanghai Realbio Technology (RBT) Co., Ltd. (http://www.realbio.cn). The experimental procedure was as follows: DNA was extracted from 371 stool samples using the MagPure Stool DNA KF Kit (Magen, China) per the manufacturer’s instructions. DNA library construction based on DNA nanospheres and shotgun metagenomic sequencing via combined probe-anchoring synthesis were performed on all the samples (MGI2000, MGI, Shenzhen, China). The overall accuracy (≥ 0.8) control strategy was used to perform quality control on the raw sequenced reads to filter out low-quality reads.

### Metagenomic classification and taxonomic analysis

The sequenced libraries were subjected to metagenomic classification using MetaPhlAn2 to obtain standard relative abundance values for species at all taxonomic levels [16]. Initially, a comparison of sequences and markers was conducted, followed by the MetaPhlAn2 classifier’s analysis of metagenomic reads against a precomputed marker catalogue via nucleotide BLAST searches. This process yielded clade abundances for one or more sequenced metagenomes. For determination of the content, the classifier was used to normalize the total reads per clade by the nucleotide length of its index, thereby providing relative abundance values for each taxonomic unit while accounting for subclade-specific indices. Microbial clades were subsequently estimated by normalizing read-based counts relative to the average genomic size of each clade.

### Untargeted analysis by ultra-performance LC-Q-TOF/MS

The analysis involved untargeted metabolomics and leveraging ultra-performance liquid chromatography–mass spectrometry (LC–MS), which involves a comprehensive workflow involving sample collection, metabolite extraction, LC–MS metabolite detection, mass spectrometry data preprocessing, multivariate statistical analysis, and differential structure identification. All the analytical procedures were performed by Shanghai Applied Protein Technology Co., Ltd. (http://www.aptbiotech.com). In the experimental process, after thawing at 4 °C, the stool samples were thoroughly mixed by shaking. Subsequently, 100 μl of each sample was transferred to a 1.5 mL EP tube. All the samples were combined with 1 mL of prechilled chloroform/methanol/water (1:2:1, v/v), followed by vortexing and mixing. The samples were then stored at 4 °C for 2 hours, followed by centrifugation at 13,000 rpm at 4 °C for 15 minutes. The resulting supernatant was filtered through a 0.22 μm filter membrane, subjected to nitrogen-assisted drying, and preserved at − 80 °C until analysis. For quality control (QC) purposes, equal amounts of each sample were synchronized and mixed to create a QC sample. The QC samples were subjected to the same chloroform/methanol/water (1:2:1, v/v) extraction process and subsequent preparation steps as were used for the individual samples. Before LC–MS mass spectrometry analysis, 100 μL of isopropanol/acetonitrile/water (2:5:3, V/V/V) was used to reconstitute each sample, and the resulting supernatant was centrifuged for subsequent detection.

### Culture and preparation of *F. prausnitzii*

The medium for *F. prausnitzii* (ATCC 27766) was LYHBHI (containing 37 g/L brain heart extract, 5 g/L yeast extract, 5 mg/L blood crystal) plus 1 g/L maltose, 1 g/L cellobiose and 0.5 g/L cysteine, and double-distilled water was added to mix the solution [17]. The bacteria were placed in an anaerobic glove box (Coy Scientific Products) with a gas concentration of 90% nitrogen, 5% hydrogen, and 5% carbon dioxide and incubated at 37 °C for 24 hours. The bacterial solution was prepared by diluting in an anaerobic glove box by centrifugation with PBS at a concentration of 2.5 × 10^9^ CFU/100 μL and then loading into an anaerobic test tube for immediate use.

### Animals

All apolipoprotein E–deficient mice were bred on a C57BL/6 background (*n* = 40). All mice were housed in a specific pathogen-free animal facility at Xinjiang Medical University. The animals were allowed access to normal food and were watered freely under a strict 12-hour light/dark cycle. All the mice in the experiment were male. The mice aged 12 weeks were given the same dose of a high-fat diet (XT108C; Jiangsu SyungPharma Biological Company, China) and randomly divided into two groups: the control group was given 0.5 mL of phosphate-buffered saline (PBS) by gavage, and the experimental group was given the same dose of *F. prausnitzii* solution diluted with centrifugal PBS. The mice were gavaged 5 times a week, always at the same time of day. All procedures conformed to the guidelines of the NIH Guide for the Care and Use of Laboratory Animals.

### In vitro culture protocols

The HCT-116 human colon carcinoma cell line was obtained from Shanghai Cell Collection (Shanghai, China). The cells were cultured in Dulbecco’s modified Eagle’s medium (DMEM) supplemented with 10% foetal bovine serum (FBS) and 1% penicillin/streptomycin (all from Biological Industries, Inc.) in a 37 °C incubator filled with 5% CO2. Cells in the logarithmic phase of growth were seeded in 12-well plates at a concentration of 1 × 10^6^ cells/mL and cultured until the cells were adherent. The cells were randomly divided into three groups: live bacteria group, inactivated bacteria group and blank control group. The term “bacteria and cell culture” refers to the Intestinal Hemi-Anaerobic Coculture System (iHACS) [18, 19]. The Transwell chamber was used, the upper chamber was closed to culture *F. prausnitzii,* and the lower chamber was inoculated with HCT-116 cells. After 24 h of treatment, proteins were extracted from HCT-116 cells by adding 200 μg/mL lipopolysaccharide (LPS) to 12-well cell culture plates.

### Assessment of atherosclerotic lesions

After the mice were anaesthetized by isoflurane, the intact aorta was dissected and opened using spring shears under a stereomicroscope (#PICO/S100, Carl Zeiss Meditec AG, Germany). The dissected aorta was rinsed in normal saline to remove residual blood from the lumen and then fixed in 4% paraformaldehyde at room temperature for 10 minutes. After rinsing in normal saline, residual oil adhering to the active lumen was removed under a microscope. The aorta was immersed in 0.3% oil red O working solution at 45 °C and incubated for 10 min in a closed chamber. Then, the aorta was differentiated in 75% alcohol until the normal aortic wall became creamy white [20]. After rinsing with normal saline, the aorta was fully spread on black absorbent paper for imaging [17].

Mouse hearts were removed, fully fixed with 4% paraformaldehyde, and dehydrated in 25% sucrose solution for more than 48 hours. The heart was removed, and Opti-mum Cutting Temperature Compound (#4583, SAKURA, USA) was added to completely soak the tissue. Five consecutive sections (10 μm thick) spanning a 550 μm long aortic sinus were collected from each mouse and stained with oil red O and haematoxylin. Images of the stained sections were obtained using an all-in-one fluorescence microscope. ImageJ software was used for the measurements.

### Immunofluorescence analysis of atherosclerotic lesions and the ileum in mice

Mouse hearts were cryosectioned. The prepared frozen sections were dried at room temperature for 15 minutes, washed three times with PBS, fixed with 4% paraformaldehyde, blocked with sheep serum and 0.3% Triton for 1 hour at room temperature, and incubated with CD68 antibody (#bs-1432R, 1:100; Bioss, China) and McP-1 antibody (#ab7202, 1:200; Abcam, UK) in a wet box at 4 °C overnight. After washing with PBS the next day, the samples were incubated with fluorescent secondary antibodies at room temperature for 1 h. The nuclei were stained with ready-to-use DAPI (#C0065, Solarbio, China) and placed in PBS at room temperature for 3 min, after which images were collected via microscopy. Two to three sheets were collected for each sample.

Mouse ileum was prepared using paraffin sectioning. The prepared paraffin sections were baked and dried, blocked with 3% hydrogen peroxide, subjected to antigen retrieval in sodium citrate buffer, blocked with goat serum for 20 minutes, and incubated with MUC-2 antibody (#DF8390, 1:50, Affinity, USA) in a wet box at 4 °C overnight. After the samples were washed with PBS the next day, fluorescent secondary antibodies were added to the samples at room temperature for 1 h. The nuclei were stained with ready-to-use DAPI and placed in PBS at room temperature for 3 min, after which the images were collected via microscopy. Three to five sheets were collected for each sample, and ImageJ software was used for analysis.

### Immunohistochemical analysis of the mouse ileum

The ileum of each mouse was prepared via paraffin sectioning. The prepared paraffin sections were baked and dried, blocked with 3% hydrogen peroxide, subjected to antigen retrieval in sodium citrate buffer, blocked with goat serum for 20 min, and incubated with a ZO-1 antibody (#AB216880, 1:100, Abcam, UK) in a wet box at 4 °C overnight. The next day, the sections were rewarmed, cleaned, and incubated with secondary antibody at 37 °C for 1 h. DAB solution was added under the microscope for 5 seconds for colour reaction, and haematoxylin staining was performed after the colour reaction. Three to five sheets were collected for each sample, and ImageJ software was used for analysis.

### Assessment of plasma biochemical parameters and cytokine levels

Animals were fasted overnight, and blood was collected in heparin-containing tubes by means of heart puncture under anaesthesia. Blood samples were centrifuged at 3000 RPM for 10 min at 4 °C and stored at − 80 °C until analysis. The plasma concentrations of creatinine (#C011–2-1, Nanjing Jiancheng, China), aminotransferase (#C009–2 and #C010–2, Nanjing Jiancheng, China), total cholesterol (#A111–1-1, Nanjing Jiancheng, China), high-density lipoprotein cholesterol (#A112–1-1, Nanjing Jiancheng, China), low-density lipoprotein cholesterol (#A113–1-1, Nanjing Jiancheng, China), and triglycerides (#A110–1-1, Nanjing Jiancheng, China) were measured enzymatically using an automated chemical analyser. The levels of plasma cytokines, including monocyte chemoattractant protein-1 (MCP-1, #SW2544505), soluble tumour necrosis factor receptor 2 (STNFR2, #SW2544507), tumour necrosis factor-α (TNF-α, #YX-201407 M), interleukin-6 (IL-6, #YX-091206 M), interleukin-1β (IL-1β, #YX-091203 M), LPS (#YX-121619 M), D-lactose (#SW2544501), and diamine oxidase (DAO) (#SW2544502), were analysed with ELISAs according to the manufacturer’s instructions (Sino Best Biological Technology Co., Ltd., China).

### Analysis of LPS levels in the faecal supernatant

Faecal samples were prefrozen at a low temperature (below − 70 °C) for more than 7 days to inactivate the eggs. Stool (0.1 g) was weighed and diluted with 0.9 mL of distilled water, thoroughly stirred and mixed, allowed to stand for 10 minutes, and centrifuged at 5000 rpm for 30 minutes, after which the supernatant was collected for testing. The levels of LPS in mouse faeces were detected via ELISAs according to the manufacturer’s instructions for LPS (#YX-121619 M; Sino Best Biological Technology Co., Ltd., China).

### qPCR of *F. prausnitzii* in faeces

Stool samples from the mice were weighed for DNA extraction. The *F. prausnitzii* primer (Supplementary Table 1) was used to amplify the target fragment. After the best target band was obtained, a DNA glue recovery kit (#B518131, Sangon Biotech, Shanghai, China) was used to recover the target fragment. The recovered target fragment was ligated and extracted with the empty plasmid, the plasmid was used as the standard material to produce the standard curve, and the quantitative detection of bacteria was carried out according to the standard curve.

### RNA extraction and real-time PCR analysis

Total RNA was extracted from the tissue using TRIzol reagent (#15596018; Thermo Scientific, MA) according to the manufacturer’s instructions. A PrimeScript reverse transcription reagent kit (#RR037A; TaKaRa, Japan) was used for cDNA synthesis. Quantitative reverse transcriptase polymerase chain reaction (RT–PCR) was performed using SYBR Premix Ex Taq (#RR820; TaKaRa, Japan) and a LightCycler® 96 System (#05815916001; Roche, Germany) according to the manufacturers’ instructions. The primers used for each specific gene are listed in Supplementary Table 1. The expression data were normalized to that of β-actin, which was used as a control housekeeping gene, and analysed according to the 2^-ΔΔCT^ method.

### Histological analysis of the mouse ileum

Ileum tissue was taken to prepare paraffin sections. The prepared paraffin sections were baked and dried, and haematoxylin and eosin were used for HE staining. The stained sections were digitally captured with an integrated fluorescence microscope, and 3–5 complete villus structures in each field were measured using ImageJ software. According to the instructions of the Alcian blue-periodic acid-Schiff (AB-PAS) staining kit (#G1285; Solarbio, China), after 10 minutes of oxidation with periodic acid, Schiff reagent was added for staining. The stained sections were digitally captured using an integrated fluorescence microscope, and the number of goblet cells per unit length was measured using ImageJ software.

### Western blotting

Protein extracts were prepared by cell lysis using radioimmunoprecipitation assay buffer. The protein concentration was determined using a BCA protein assay kit, and 20 mg of protein extract was separated via 10% sodium dodecyl sulphate–polyacrylamide gel electrophoresis followed by transfer onto a polyvinylidene fluoride microporous membrane. The membrane was blocked with 5% nonfat milk at room temperature for 2 h and incubated with rabbit primary antibodies against ZO-1 (#ab216880, 1:1000; Abcam, UK) at 4 °C overnight. After incubation with goat anti-rabbit secondary antibody at room temperature for 2 h, the density of the protein band was analysed using ImageJ.

### Statistical analysis

Statistical analyses were conducted using SPSS version 22 (SPSS, Inc., Chicago, IL, USA) and R version 3.2.4 (R Foundation for Statistical Computing, Vienna, Austria). The bioinformatics methods used are detailed in the Supplementary materials. The Shapiro–Wilk test was used to determine the normality of the data. Normally distributed data are presented as the mean ± SEM or mean ± SD, and nonnormally distributed data are presented as the median ± interquartile range (25th to 75th percentiles). Two-group differences were assessed with the 2-tailed Student’s t test for normally distributed data and the Mann–Whitney U test for non-normally distributed data. Categorical variables were compared using Fisher’s exact test or the χ2 test, with *P* <  0.05 indicating statistical significance. For the mouse study, the Kolmogorov–Smirnov test was used to assess sample distribution. Parametric data were analysed using a two-tailed unpaired Student’s t test for two-group comparisons and one-way ANOVA with Bonferroni post hoc correction for more than two groups. Nonparametric data were analysed with the Mann–Whitney U test. Fisher’s exact test was used to evaluate the incidence of aneurysm formation and rupture, and the log-rank test was used to assess the cumulative stroke incidence. *P* <  0.05 was considered to indicate statistical significance in all analyses.

## Results

### Significant differentially abundant Bacteria in CAD patients with varying disease severities

**Fig. 1:**
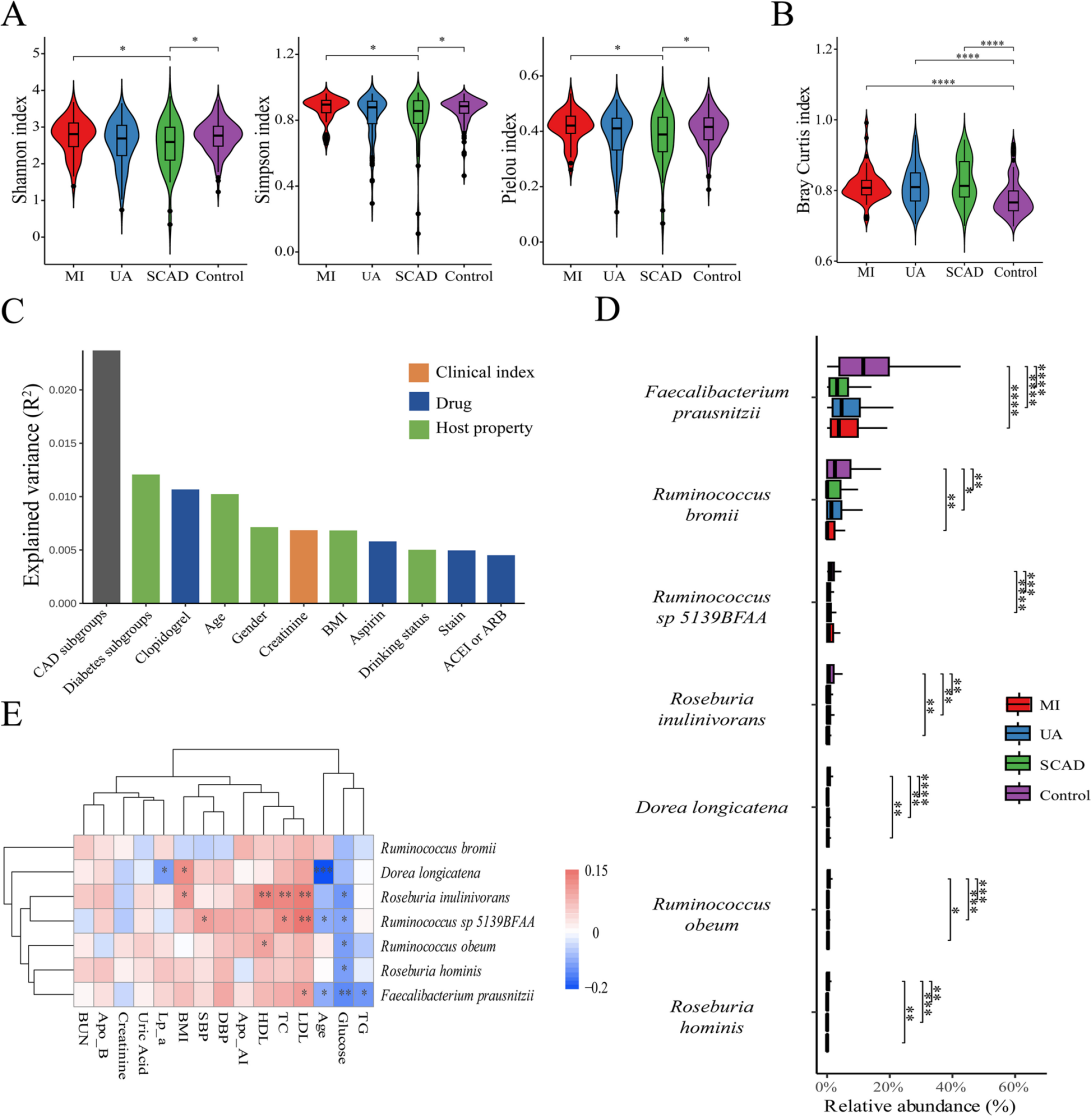
Gut Microbiota Diversity Analysis. **a** α-Diversity of the gut microbiota at the species level. **b** β-Diversity of the gut microbiota at the Bray–Curtis distance species level. **c** Indices affecting the differences in the gut microbiota (*p* <  0.05) and corresponding contributions (R^2^). **d** Differential gut microbiota observed among the various groups. E: Heatmap of the correlation between differentially abundant gut microbiota constituents and clinical indicators. *, *P* <  0.05; **, *P* <  0.01; ***, *P* <  0.001; ****, *P* <  0.0001

In this study, a cohort of 371 participants was enrolled, comprising 215 patients with CAD, 56 with SCAD, 106 with UA, 53 with MI, and 156 controls without CAD. The baseline characteristics of these participants are detailed in Table 1. Metagenomic sequencing was performed on faecal samples from all subjects and revealed notable differences in alpha and beta microbial diversity at the species level across the various patient groups (Fig. 1a-b). Factors such as population characteristics can influence the composition of the gut microbiota [21, 22]. Consequently, an Adonis analysis was performed to determine which specific baseline characteristics most significantly affected gut microbiota diversity, and the extended findings are shown in Supplementary Table 2 [23]. The results highlighted that coronary artery disease had the most significant influence on the variations in the gut microbiota (Fig. 1c).

**Table 1:** Characteristics of the study cohort

Variable	MI (***n*** = 53)	UA (***n*** = 106)	SCAD (***n*** = 56)	Control (***n*** = 156)	*P* value
Age, yr *	60 ± 12	60 ± 11	60 ± 10	57 ± 11	0.56
**Male,n** ^**#**^	43 (81.13%)	70 (66.04%)	33 (58.93%)	77 (49.36%)	< 0.001 ^a b^
**Body mass index, kg/m**^**2+**^	25.69 (3.81)	25.14 (5.1)	25.15 (4.84)	25 (5.48)	0.828
**Systolic blood pressure, mmHg** ^**+**^	128 (22)	131 (24)	124 (27)	124 (22)	0.021 ^b^
**diastolic blood pressure, mmHg** ^**+**^	77 (16)	76 (16)	73 (17)	75 (12)	0.51
**Smoking status**					< 0.001^a e^
Never or Past ^**#**^	31 (58.49%)	62 (58.49%)	38 (67.86%)	127 (81.41%)	
Current ^**#**^	22 (41.51%)	44 (41.51%)	18 (32.14%)	29 (18.59%)	
**Drinking status**					< 0.001^a b^
Non-heavy Drinker ^**#**^	25 (47.17%)	73 (68.87%)	38 (67.86%)	123 (78.85%)	
Heavy Drinker ^**#**^	28 (52.83%)	33 (31.13)	18 (32.14%)	33 (21.15%)	
**Basic diseases**					
Hypertension ^**#**^	35 (66.04%)	63 (59.43%)	37 (66.07%)	65 (41.67%)	0.001^a b c^
Diabetes ^**#**^	23 (43.4%)	34 (32.08%)	17 (30.36%)	21 (13.46%)	< 0.001^a b c^
**Long term oral medication**					
Aspirin ^**#**^	46 (86.79%)	88 (83.02%)	42 (75%)	60 (38.46%)	< 0.001^a b c^
Clopidogrel ^**#**^	33 (62.26%)	69 (65.09%)	37 (66.07%)	8 (5.13%)	< 0.001^a b c^
Stain ^**#**^	44 (83.02%)	81 (76.42%)	47 (82.93%)	67 (42.95%)	< 0.001^a b c^
β-block ^**#**^	38 (71.7%)	64 (60.38%)	34 (60.71%)	46 (29.49%)	< 0.001^a b c^
Calcium Channel Blockers ^**#**^	8 (15.09%)	26 (24.53%)	17 (30.36%)	29 (18.59%)	0.155
ACEI or ARB ^**#**^	25 (47.17%)	37 (34.91%)	26 (46.43%)	35 (22.44%)	0.001^a c^
**Laboratory results**					
Blood Urea Nitrogen, mmol/L ^**+**^	5.05 (2.73)	5.11 (2.1)	5.4 (1.4)	5.05 (1.8)	0.26
Creatinine, umol/L ^**+**^	71.5 (20.28)	66 (23)	62 (22.84)	62 (19)	< 0.001^a d e^
Uric Acid, umol/L ^**+**^	333 (141.25)	308 (109)	327 (123)	310 (111)	0.111
Glucose, mmol/L ^**+**^	5.29 (2.87)	5.19 (2.07)	4.84 (1.9)	4.76 (0.82)	< 0.001^a b^
Triglyceride, mmol/L ^**+**^	1.48 (1.03)	1.37 (0.99)	1.57 (1.18)	1.32 (0.88)	0.114
Total cholesterol, mmol/L ^**+**^	3.47 (1.36)	3.34 (1.26)	3.42 (1.29)	3.96 (1.18)	0.001^b^
High Density Lipoprotein, mmol/L ^**+**^	1.04 (0.31)	1.02 (0.39)	1.06 (0.41)	1.14 (0.46)	0.002^a b^
Low Density Lipoprotein, mmol/L ^**+**^	2.09 (1.31)	2.03 (1.05)	1.84 (1.17)	2.53 (1.06)	< 0.001^b c^
Apolipoprotein AI, g/L *	0.99 ± 0.22	1.0 ± 0.33	1.1 ± 0.29	1.1 ± 0.28	0.002^a b^
Apolipoprotein B, g/L *	0.91 ± 0.31	0.87 ± 0.29	0.89 ± 0.31	0.96 ± 0.29	0.08
Lipoprotein(a), mg/L ^**+**^	195.02 (247.72)	125.89 (245.44)	123.2 (161.8)	105.35 (180.23)	0.124

Continuous, normally distributed variables among the four groups were analysed by a one-way analysis of variance. The Kruskal-Wallis H-test was applied for data of this type that were not normally distributed. Continuous, normally distributed variables between two groups were analysed by Student’s t-test. The MannWhitney U test was applied for data of this type that were not normally distributed. Categorical variables were compared by the χ2 test. N/A not available. Drinking history is defined as patients who consumed ≥50 g of alcohol per day

^**+**^ median (IQR), * mean ± SD, ^**#**^ n (%)

^a^*P* <  0.05 for equality between MI vs. control. ^b^*P* <  0.05 for equality between UA vs. control. ^c^*P* <  0.05 for equality between SCAD vs. control. ^d^*P* <  0.05 for equality between SCAD vs. MI. ^e^*P* <  0.05 for equality between UA vs. MI

For precise species-level analysis, Kruskal–Wallis tests were employed in conjunction with aldex2 and Bonferroni–Holm correction, leading to the identification of significant differences in the microbiota (Fig. 1d) [24]. According to the results, six microbiota species, *F. prausnitzii*, *Ruminococcus bromii*, *Ruminococcus* sp. *5139BFAA*, *Roseburia inulinivorans*, *Dorea longicatena*, *Ruminococcus obeum*, and *Roseburia hominis*, exhibited significant differences across groups, with *F. prausnitzii* being the most pronounced. To further delineate the specific causative bacteria involved, we conducted a propensity score matching analysis of the population considering the factors influencing the gut microbiota. Postanalysis of the differential bacteria revealed the consistent presence of *F. prausnitzii*, *R. bromii*, and *R. inulinivorans* across the four groups (Fig. 1a in the online-only Data Supplement). Interestingly, when different groups were compared based on their medication history of oral aspirin or clopidogrel, the Bonferroni–Holm adjustment was used to identify differential bacteria, and no statistically significant differences were found. Finally, we examined the correlations between these diverse microbiota constituents and clinical parameters (Fig. 1e and b in the online-only Data Supplement).

### Cluster analysis and diagnostic efficacy of CAD for patients with different disease severities in the gut microbiota

**Fig. 2:**
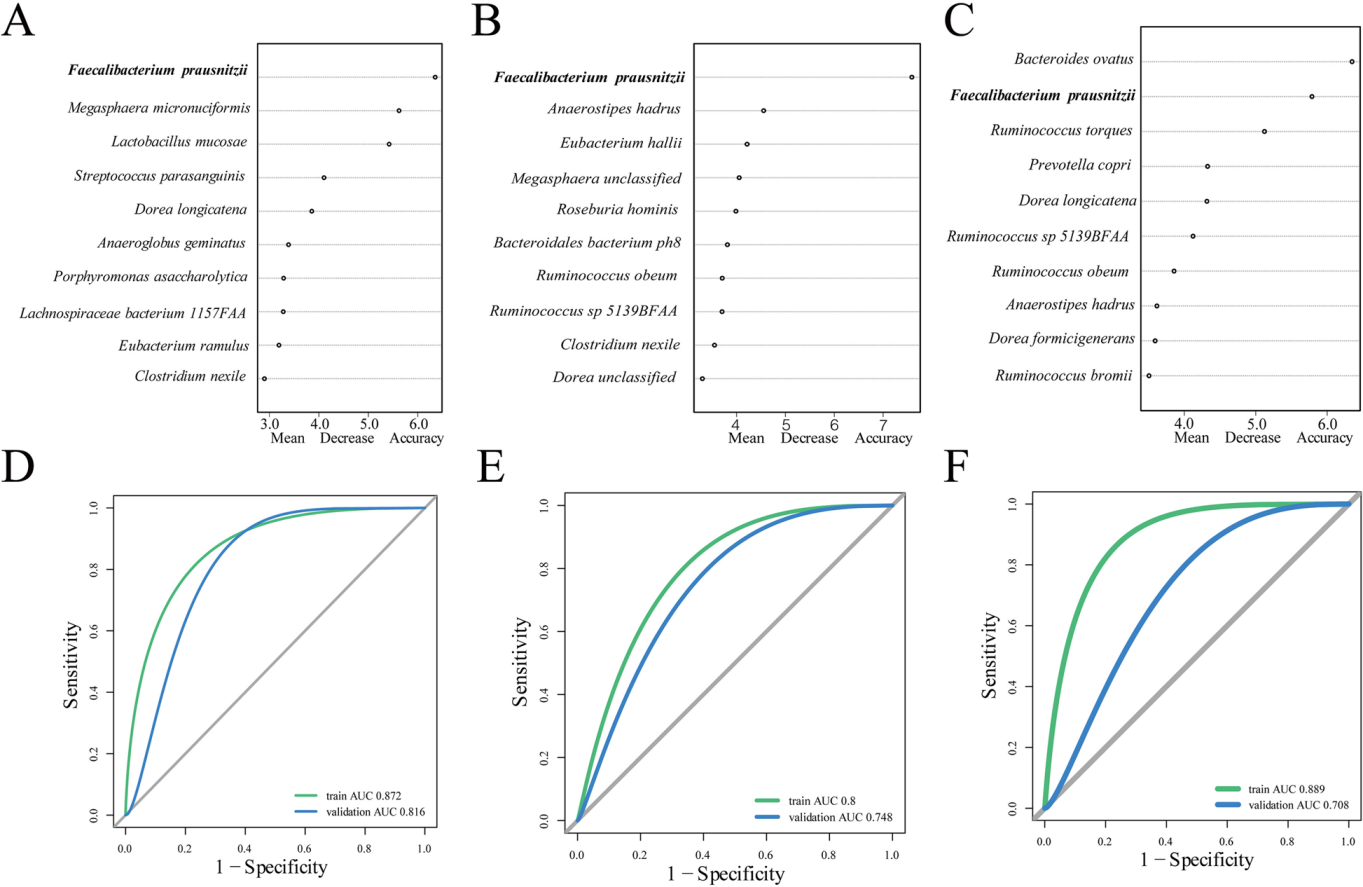
Important Biomarkers of Gut Microbes. The included population was randomly divided into a training cohort and a validation cohort. With respect to the training cohort, the random forest model was used to search for important markers. **a**, **b**, and **c** represent the top 10 important gut microbiota constituents in the MI group and the control group, the UA group and the control group, and the SCAD group and the control group, respectively. **d**, **e**, and **f** are the ROC curves for different groups of important gut microbiota constituents in the training cohort and the validation cohort, with the green curve representing the training cohort and the blue curve representing the validation cohort

To better understand the dominant microbiota in the population, we performed cluster analysis on 371 samples [25]. At the genus level, the total sample could be clustered into three genera: *Bacteroides*, *Prevotella*, and *Megamonas* (Fig. 2a in the online-only Data Supplement). After clustering, there was no significant difference in sample size between the three groups with different types of coronary artery disease and the control group (Fig. 2b in the online-only Data Supplement). A Venn diagram was drawn for the genus-level microbiota of the four groups, and the control group had more independent strains than the other three groups did (Fig. 2c in the online-only Data Supplement). For further investigation, microbiota abundance at the species level was used for clustering, and the bacteria were clustered into five groups: *F. prausnitzii, Prevotella copri, Megamonas unclassified, Escherichia coli, and Bacteroides fragilis* (Fig. 2d in the online-only Data Supplement). Subsequently, the MI, UA, SCAD and control groups were assessed separately for each clustered group. We found that in the *F. prausnitzii* cluster, the number of patients in the control group without coronary artery disease was significantly greater than that in the other four groups (Fig. 2e in the online-only Data Supplement). The annotated species-level microbiota are displayed in a Venn diagram (Fig. 2f in the online-only Data Supplement).

We randomly divided the total cohort into a training cohort and a validation cohort at an approximately 3:1 ratio. For the validation cohort, we selected 50 healthy controls and 50 patients with CAD, comprising 15 patients with SCAD, 28 with UA, and 7 with MI. The species-level microbiota were cross-checked 10-fold to identify more important characteristic species in the training cohort through the random forest model. The important markers of different types of CAD populations compared with those of the control group were identified. Interestingly, *F. prausnitzii* was found to be highly important in both groups (Fig. 2a-c). Furthermore, the important gut microbiota identified in the training cohort demonstrated significant predictive capability for various types of CAD in both the training and validation cohorts. Specifically, for MI, the area under the curve (AUC) was 0.872 in the training cohort and 0.816 in the validation cohort. For UA, the values were 0.8 in the training cohort and 0.748 in the validation cohort. For the SCAD cohort, the area under the curve (AUC) was 0.889 in the training cohort and 0.708 in the validation cohort. These results indicate that the gut microbiota could effectively predict the occurrence of various types of CAD (Fig. 2d-f).

### Metabolomic signatures of CAD patients with different disease severities

**Fig. 3:**
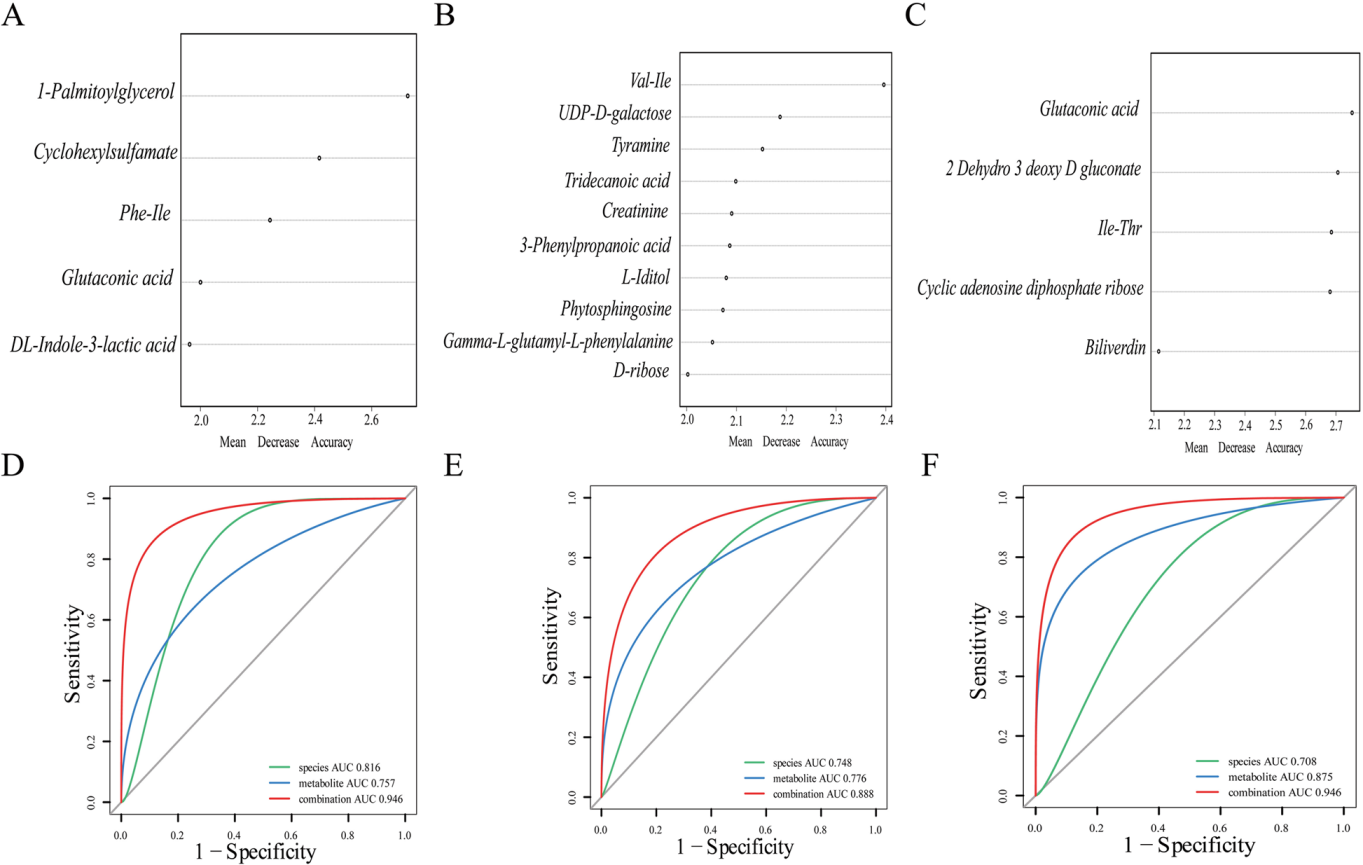
Important Metabolite Biomarkers. The first 5–10 important metabolites between different groups were screened in the validation cohort. **a**, **b**, and **c** represent the MI and control groups, the UA and control groups, and the SCAD and control groups, respectively. **d**-**f** In the verification cohort, important gut microbiota and intestinal metabolites were used to construct prediction models of disease between different groups. The green curve represents the gut microbiota, and the blue curve represents intestinal metabolites. The red curve represents the combined analysis of the above two objects

The faecal samples from the selected validation cohort were subjected to untargeted metabolite testing. Variable importance for the projection (VIP) values were utilized to screen metabolites; metabolites with a VIP > 1 according to multidimensional statistical analysis and a *P* value < 0.05 according to univariate statistical analysis were considered to be significantly different. Among the four groups, namely, MI patients, SCAD patients with less severe CAD, controls, and others, 83 distinct metabolites were identified for both anions and cations (Table 3 in the online-only Data Supplement). Subsequently, the random forest model was used to identify important metabolites, and their efficacy was assessed using the area under the ROC curve. These results were subsequently compared with the diagnostic efficacy of important microbiota constituents, and combined diagnosis was explored. The results revealed that, compared to that in the control group, the AUC for the gut microbiota in the MI group was 0.816; for the metabolites, it was 0.757; and for the combined AUC, it was 0.946. In the UA group, the AUC for the gut microbiota was 0.748; for the metabolites, it was 0.776; and for the combined AUC, it was 0.888. For the SCAD group, the gut microbiota AUC was 0.708, the metabolite AUC was 0.875, and the combined AUC reached 0.946 (Fig. 3a-f). These results indicate differential diagnostic capabilities of microbiota and metabolites across the MI, UA, and SCAD groups, with the combined approach showing enhanced diagnostic efficiency.

**Fig. 4:**
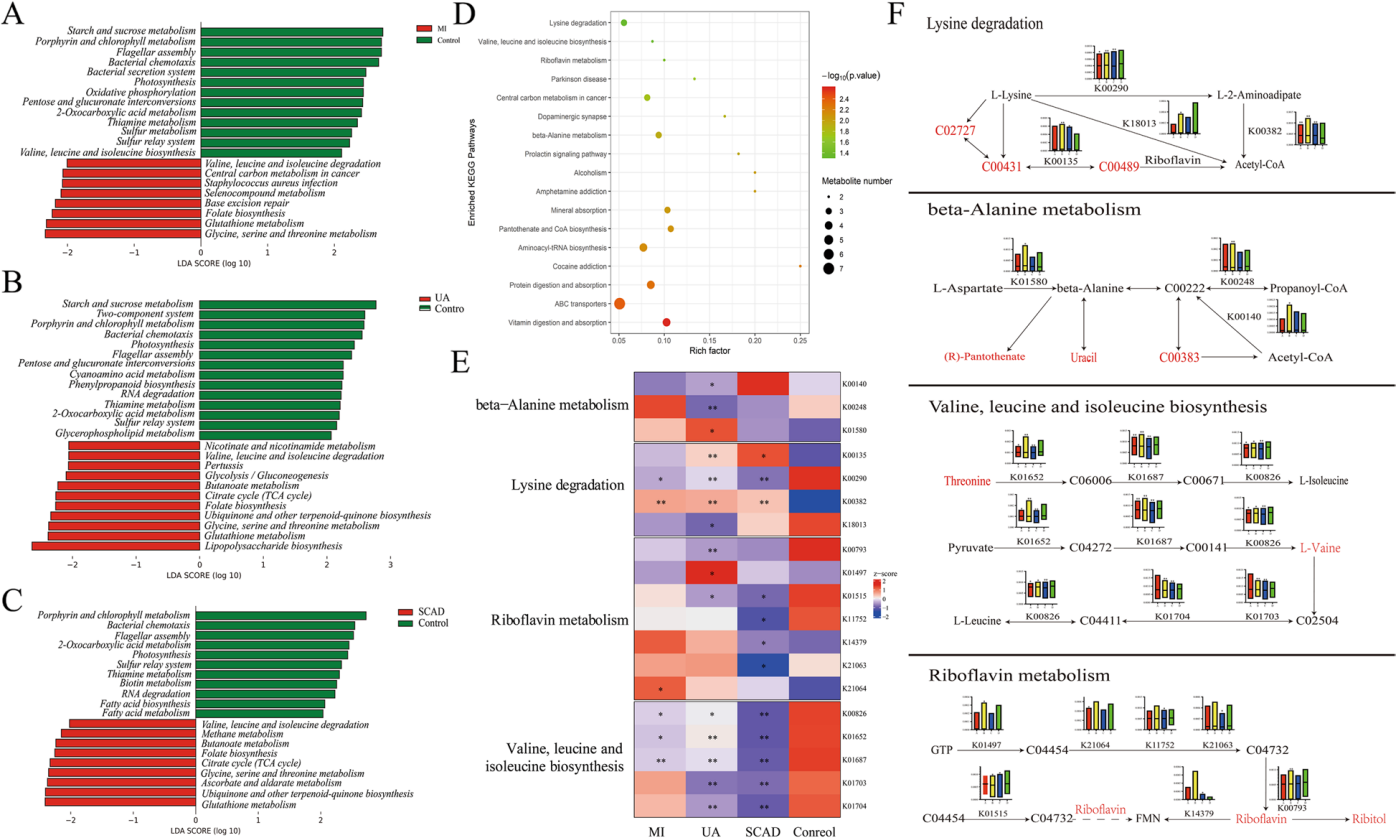
CAD-associated changes in gut microbial function and their metabolomic associations. **a** Comparisons of the relative abundance of KEGG modules between the MI group and control group. **b** Comparisons of the relative abundance of KEGG modules between the UA group and control group. **c** Comparisons of the relative abundance of KEGG modules between the SACD group and control group. **d** Bubble chart of metabolite annotation-related pathways. **e** The abundance of KO genes involved in representative pathway modules was significantly different in at least one CAD severity group. KO genes with a prevalence of 5% or higher are shown. Significant changes are denoted as follows: *, P <  0.05; **, P <  0.01 according to the Wilcoxon rank sum test. **f** Changes in gene abundance in (**e**) are shown in a simplified pathway presentation

Furthermore, a comprehensive prediction analysis combining metagenomic and metabolomic functions was performed. First, the metagenomic gene fragments were annotated with relevant pathways in KEGG and compared with those of the control group for different degrees of CAD (Fig. 4a-c). Subsequently, the metabolites were annotated to relevant pathways to construct a metabolite pathway bubble map (Fig. 4d). The influence of the gut microbiota on the development of diseases should be the result of the combined effects of the microbiota and metabolites. To better conduct joint prediction analysis, we conducted joint analysis of metagenomic gene segments and metabolite pathways and found that intestinal microorganisms could play specific roles in the metabolism of these metabolites [26]. According to the annotation results, the microbiota can participate in the gut metabolite pathway, and they may jointly affect amino acid metabolism and vitamin metabolism (Fig. 4e-f).

To determine which substances are most important for the occurrence and development of CAD, the diagnostic efficacy of the important metabolites and microbiota identified by the model were evaluated individually. A total of 371 samples were used for gut microbiota assessment, and 100 samples were used for gut metabolite assessment. The final results showed that *F. prausnitzii* exhibited better diagnostic efficacy in the comparison between different degrees of CAD and the control group, as detailed in Table 2. Taken together, these results suggest that *F. prausnitzii* has potential antiatherosclerotic effects.

**Table 2:** Evaluating Biomarkers in Identifying CAD Severity

Biomarker	Comparisons of different CAD severities								
	MI VS Control			UA VS Control			SACD VS Control		
	AUC	***P*** value	95% CI	AUC	***P*** value	95% CI	AUC	P value	95% CI
**Gut bacterial species**									
Faecalibacterium_prausnitzii	0.728	< 0.001	0.647–0.81	0.717	< 0.001	0.645–0.79	0.78	< 0.001	0.703–0.856
Dorea_longicatena	0.659	0.002	0.565–0.753				0.746	< 0.001	0.659–0.833
Eubacterium_ramulus	0.654	0.003	0.556–0.753						
Lachnospiraceae_bacterium_1_1_57FAA	0.664	0.001	0.575–0.752						
Lactobacillus_mucosae	0.646	0.004	0.544–0.747						
Megasphaera_micronuciformis	0.607	0.036	0.502–0.712						
Eubacterium_hallii				0.646	0.001	0.564–0.728			
Roseburia_hominis				0.669	< 0.001	0.59–0.748			
Anaerostipes_hadrus				0.633	0.002	0.55–0.716	0.681	0.001	0.581–0.78
Ruminococcus_obeum				0.648	0.001	0.5690.728	0.716	< 0.001	0.626–0.806
Ruminococcus_sp_51_39BFAA				0.669	< 0.001	0.588–0.75	0.751	< 0.001	0.659–0.844
Bacteroides_ovatus							0.731	< 0.001	0.637–0.824
Dorea_formicigenerans							0.628	0.017	0.527–0.728
Ruminococcus_bromii							0.698	< 0.001	0.603–0.792
Ruminococcus_torques							0.699	< 0.001	0.594–0.804
**Fecal metabolites**									
Cyclohexylsulfamate	0.763	0.025	0.512–1						
1-Palmitoylglycerol	0.831	0.005	0.677–0.986						
Phe-Ile	0.763	0.025	0.56–0.966						
2-Dehydro-3-deoxy-D-gluconate							0.712	0.013	0.549–0.875
Ile-Thr							0.688	0.028	0.511–0.865

### Gavage with *F. prausnitzii* attenuated the formation of atherosclerotic plaques in mice

**Fig. 5:**
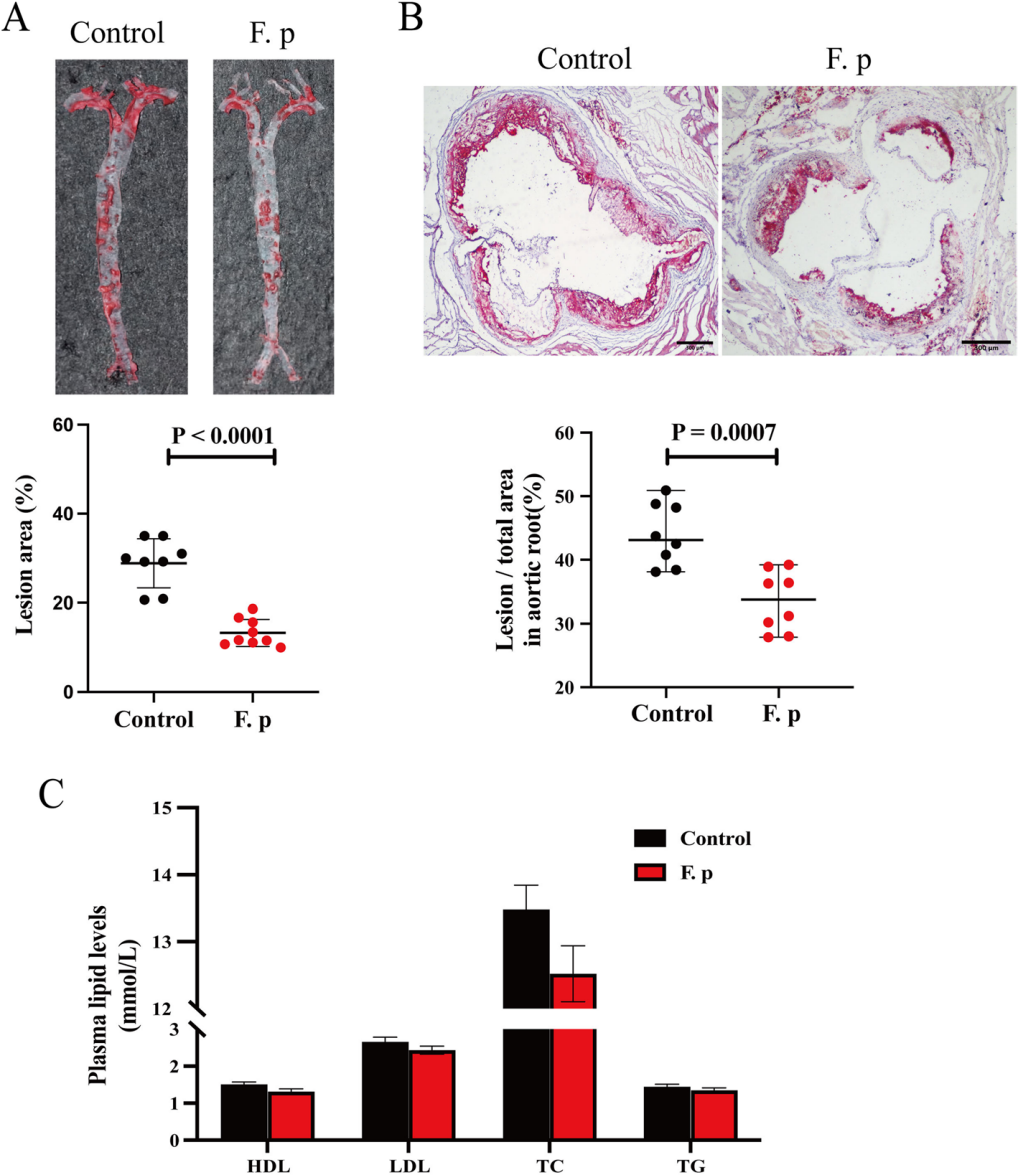
*F. prausnitzii* attenuates atherosclerotic lesions. **a** Representative photomicrographs of oil red O staining and quantitative analysis of the atherosclerotic lesion area in the aortas (8 samples per group). **b** Representative image of oil red O staining and quantitative analysis of the atherosclerotic lesion area in the aortic sinus (8 samples per group). The black bar represents 500 μm. **c** Comparison of plasma lipid profiles (12 to 15 samples per group). The data are presented as the mean ± standard error of the mean. All *P* values were determined by two independent sample t tests

To verify the antiatherosclerotic effect of *F. prausnitzii*, we first simulated the survival of *F. prausnitzii* via a digestive tract assay. The results suggest that *F. prausnitzii* could be used as an experimental agent in vivo (Fig. 3a-b in the online-only Data Supplement). Subsequently, 12-week-old male APOE^−/−^ mice were randomly assigned to two groups. *F. prausnitzii* was cultured for 24 hours and subsequently precipitated through centrifugation. The precipitated culture was diluted to 2.5 × 10^9^ CFU/100 μL using PBS. The experimental group was given bacterial solution by gavage, while the control group was given PBS gavage 5 times a week for 12 weeks. During this period, body weight was measured every 2 weeks, and a comparison showed that there was no significant difference between the two groups (Fig. 4a in the online-only Data Supplement). After a 12-week period, the mice were euthanized under anaesthesia using isoflurane. Subsequently, the livers and spleens of the mice were collected for comparison of organ indices, revealing that there was no hepatosplenomegaly in the experimental group. (Fig. 4b-c in the online-only Data Supplement). Moreover, plasma was taken for liver and kidney function tests, and the experimental group given *F. prausnitzii* did not exhibit abnormal liver and kidney function compared with the control group (Fig. 4d-f in the online-only Data Supplement). After evaluation of the mouse model, the atherosclerotic indices were detected. Compared with those in the control group, the gross aorta and aortic root plaque tissue in the experimental group were significantly reduced (Fig. 5a-b). Plasma lipid levels (Fig. 5c) and fasting blood glucose levels (data not shown) were not significantly different between the experimental group and the control group. Therefore, we can confirm that *F. prausnitzii* has an antiatherosclerotic effect and that this effect is independent of blood lipids and blood glucose.

### Treatment of local inflammation of the aorta and systemic inflammation via gavage with *F. prausnitzii*

**Fig. 6:**
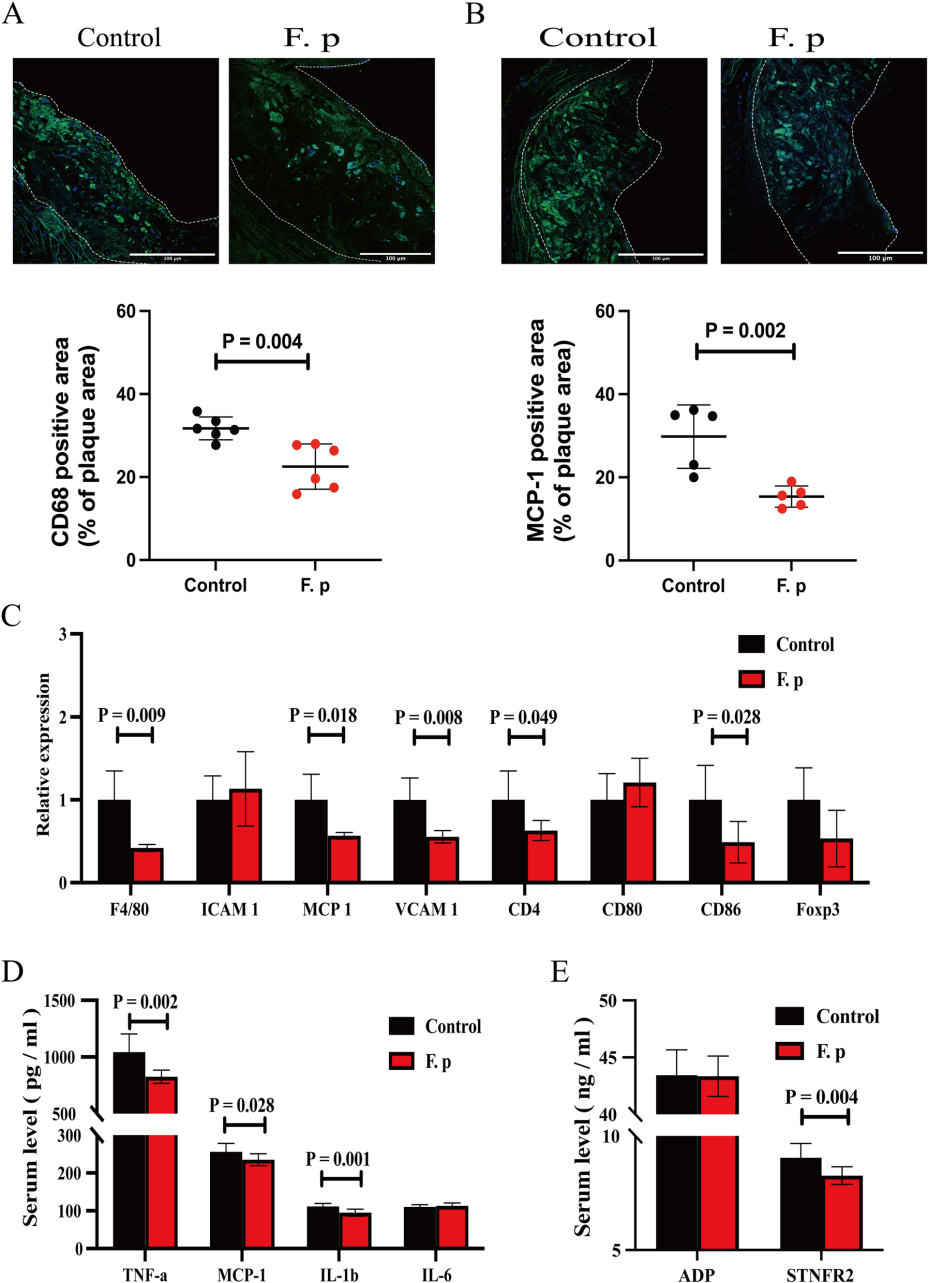
*F. prausnitzii* attenuates local and systemic inflammation. **a** Representative fluorescence staining of macrophages and quantitative analysis of CD68-positive staining in the aortic sinus (6 samples per group). **b** Representative fluorescence staining of macrophages and quantitative analysis of MCP-1–positive staining in the aortic sinus (5 samples per group). **c** mRNA levels in the atherosclerotic aorta. The data were normalized to the housekeeping gene β-actin (6 samples per group). **d** and **e** The circulating levels of TNF-α, MCP-1, interleukin-1β (IL-1β), interleukin-6 (IL-6), soluble tumour necrosis factor receptor II (sTNFR II), and adiponectin (ADP) were measured via ELISAs (10 samples per group). The data are presented as the mean ± standard error of the mean. All *P* values were determined by two independent sample t tests

To determine the reasons for the antiatherosclerotic effects of *F. prausnitzii*, we performed a quantitative assessment of the aortic roots using immunofluorescence analysis, which revealed significant differences in the expression of macrophage markers and macrophage chemokines, with *P* values of 0.004 and 0.002, respectively (Fig. 6a-b). Subsequently, we performed quantitative RT–PCR assays to assess the mRNA expression of immune cell markers, chemokines, and chemokine receptors in the aortas of atherosclerotic mice. In the experimental group, compared to those in the control group, the expression of macrophage markers, chemokines, and chemokine receptors was lower in the atherosclerosis group (Fig. 6c). Local inflammation in the aorta is the main reason for the acceleration of atherosclerosis. These results suggest that *F. prausnitzii* may protect against atherosclerosis by reducing inflammation. To further test this hypothesis, we assessed systemic inflammatory responses via the use of plasma inflammatory cytokines. The plasma levels of proatherogenic cytokines in the experimental group were significantly lower than those in the control group (Fig. 6d-e). In conclusion, these data suggest that *F. prausnitzii* can play an antiatherogenic role by reducing the systemic inflammatory response and inhibiting local inflammation, which is mainly caused by aortic macrophages.

### Gavage with *F. prausnitzii* alters the gut microbial environment

*F. prausnitzii* can produce butyrate in the intestine, which has anti-inflammatory effects [3, 4]. To determine whether *F. prausnitzii* acted through butyrate, we collected faeces of mice in both groups before sacrifice for targeted short-chain fatty acid assays. Surprisingly, the levels of short-chain fat dominated by butyrate were significantly lower in the experimental group than in the control group according to gut microbiota metabolite analysis, suggesting that the anti-inflammatory effect of *F. prausnitzii* was not mediated by butyrate (Fig. 5 in the online-only Data Supplement).

**Fig. 7:**
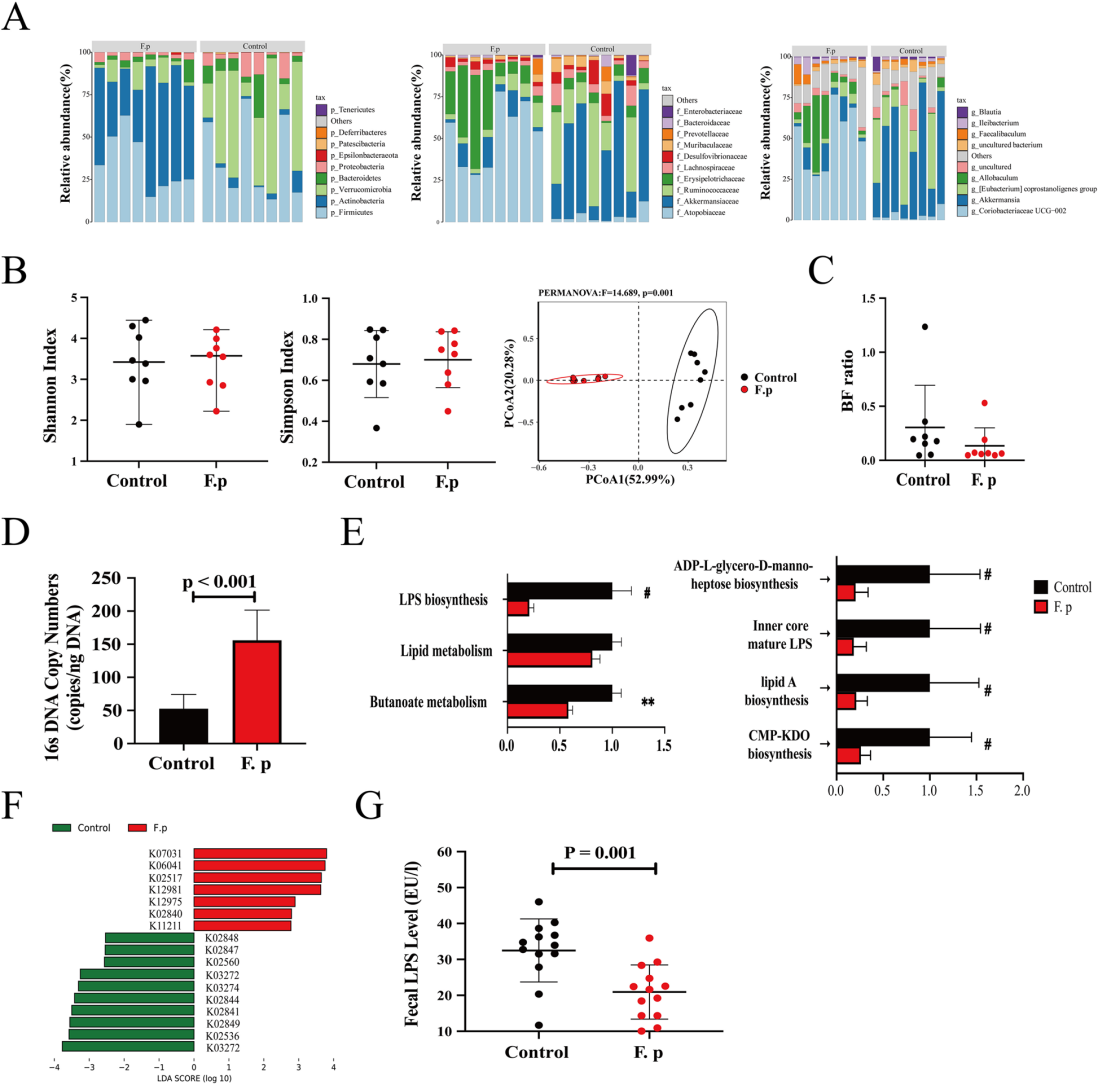
Gavage with *F. prausnitzii* alters the gut microbial environment (8 samples per group). **a** Stacking map of the percentage of each species level. **b** Alpha and beta diversity. **c** Bacteroidetes-to-Firmicutes (B F) ratio. **d** Quantification of *F. prausnitzii* in faeces by qPCR. **e** Comparison of related pathways according to 16S ribosomal RNA sequencing data analysed using PICRUSt. #:< 0.0001; **:< 0.001. F: Analysis of the KO genes in the LPS pathway. **g** Faecal LPS levels were detected via ELISAs (13 samples per group). The data are presented as the mean ± standard error of the mean. All P values were determined by two independent sample t tests. **: < 0.01, ^#:^ < 0.0001

To further elucidate the underlying mechanisms of the anti-inflammatory effects of *F. prausnitzii*, we conducted 16S rRNA gene sequencing of mouse faecal samples, which revealed alterations in the composition of the gut microbiota in mice following bacterial gavage (Fig. 7a-b). The Bacteroidetes/Firmicutes ratio did not significantly differ between the two groups (Fig. 7c). Compared with that in the control group, the faecal *F. prausnitzii* content in the mice was significantly greater after gavage (Fig. 7d). Notably, the prediction of bacterial gene functions based on 16S rRNA gene sequences using PICRUSt revealed some functional diversity among the two groups.

*F. prausnitzii* is a clear producer of butyrate in the intestine, and gut microbial function predictions were consistent with short-chain fatty acid (SCFA) metabolite analysis, which revealed that butyrate production was reduced in the experimental group (Fig. 7e). There was no significant difference in lipid metabolism between the experimental group and the control group, but the LPS synthesis pathway was significantly inhibited. Further analysis of LPS synthesis showed that lipid A and L-glycero-D-manno-oct-2-ulosonic acid (3-deoxy-D-manno-heptose, L-glycero-D-manno-heptose) synthesis in the LPS synthesis pathway was inhibited in the experimental group. In addition, LPS maturation was significantly inhibited. Finally, we performed differential analysis of genes involved in the LPS synthesis pathway and found significant differences in major synthesis genes between the two groups. To verify the 16S rRNA gene sequencing results, we determined the LPS content in mouse faeces. Compared to those in the control group, which was administered PBS via gastric gavage, the faecal LPS levels in the experimental group that received *F. prausnitzii* through gastric gavage were significantly lower (Fig. 7g).

### Gavage with *F. prausnitzii* ameliorated endotoxaemia and strengthened tight junction formation

**Fig. 8:**
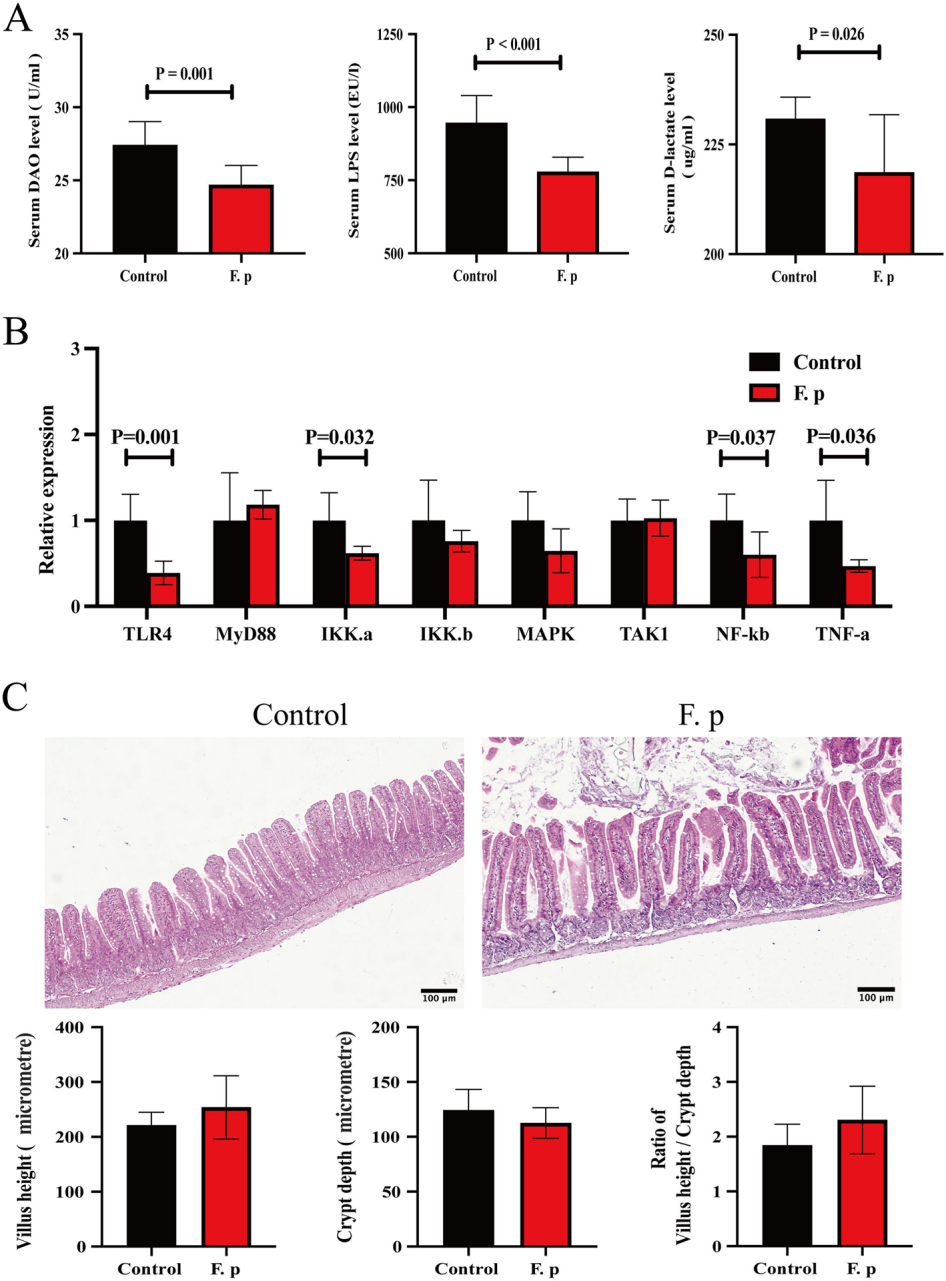
Gut barrier function between the intervention group and control group. **a** The circulating levels of D-lactate, lipopolysaccharide (LPS) and diamine oxidase (DAO) were measured via ELISAs (9 samples per group). **b** mRNA levels in the atherosclerotic aorta. The data were normalized to the housekeeping gene β-actin (5 samples per group). **c** Representative haematoxylin-eosin staining of the ileum and quantitative analysis of haematoxylin-eosin staining in the ileum (7 samples per group). The black bar represents 100 μm. The data are presented as the mean ± standard error of the mean. All P values were determined by two independent sample t tests

Endotoxaemia can cause systemic inflammation and aggravate atherosclerosis [27, 28]. Since LPS synthesis is primarily derived from the gut, plasma LPS levels are also an important measure of intestinal barrier function [29, 30]. In the present study, compared with those in the control group, the intestinal barrier indices, including plasma DAO, LPS and D-lactose levels, were lower in the experimental group, with *P* values of 0.026, < 0.001 and 0.001, respectively (Fig. 8a). To further elucidate the relationship between *F. prausnitzii* and LPS, we assessed the abundance of *F. prausnitzii* and the faecal and plasma LPS levels in the experimental group. Our findings demonstrated a notable negative correlation between the abundance of *F. prausnitzii* and the LPS concentration (Fig. 6a-b in the online-only Data Supplement). In conclusion, mice treated with *F. prausnitzii* by gavage exhibited enhanced intestinal barrier function and decreased intestinal permeability, resulting in decreased plasma LPS levels. Plasma LPS exacerbates atherosclerosis through the activation of the Toll-like receptor 4 signalling pathway in a dose-dependent manner [31]. These results were further validated in mouse aortae. Compared with that in the control group, the expression of important genes related to signalling pathways was decreased in the experimental group (Fig. 8b). In conclusion, a reduction in LPS levels and a change in intestinal permeability are the key factors leading to a reduction in endotoxaemia, and a reduction in endotoxaemia is the mechanism by which *F. prausnitzii* acts against atherosclerosis.

**Fig. 9:**
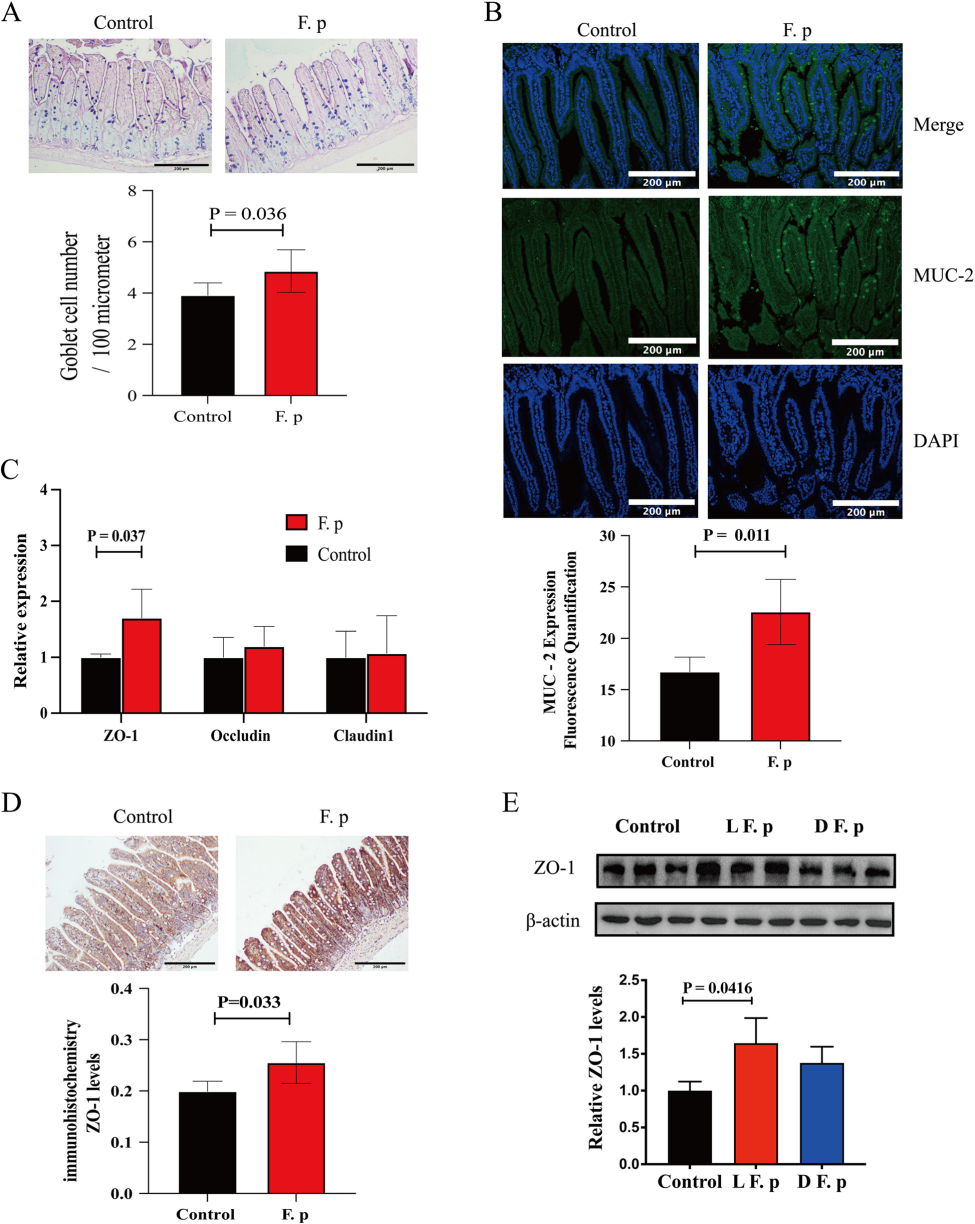
Intestinal mucus barrier and mechanical barrier properties of the intervention group and control group. **a** Representative Alcian blue-periodic acid-Schiff staining of the ileum and quantitative analysis of Alcian blue-periodic acid-Schiff staining in the ileum (6 samples per group). **b** Representative sections and quantitative analysis of MUC-2 in the small intestine (5 samples per group). **c** mRNA levels in the small intestine. The data were normalized to the housekeeping gene β-actin (5 samples per group). **d** Representative sections and quantitative analysis of ZO-1 in the small intestine (5 to 6 samples per group). **e** Comparison of ZO-1 protein levels among the live *F. prausnitzii* group (LF. p), inactivated *F. prausnitzii* (DF. p) group and control group after cell model intervention. The white/black bar represents 500 μm. The data are presented as the mean ± standard error of the mean. All P values were determined by two independent sample t tests

To further validate the changes in intestinal barrier function, we conducted HE staining of the ileum to assess villus length, crypt depth, and the villus-to-crypt ratio. Interestingly, there were no significant differences in villus length, crypt depth, or the villus-to-crypt ratio between the experimental and control groups (Fig. 8c). Intestinal barrier function is related not only to intestinal structural integrity but also to the intestinal mucosal barrier and mechanical barrier. Therefore, AB-PAS staining was performed to evaluate mucus-producing goblet cells. Compared with that in the control group, the number of goblet cells in the experimental group was greater (Fig. 9a). The Muc-2 protein is the main component of the mucus layer, so immunofluorescence staining was performed for quantitative assessment. Compared with that in the control group, the expression of the MUC-2 protein was increased in the experimental group (Fig. 9b). Intestinal permeability is regulated by the mucus layer and intestinal tight junctions. The former is the first line of defence against bacterial adhesion, while the latter further blocks the invasion of pathogens and bacterial products [32, 33]. To further study intestinal tight junctions, we performed quantitative RT–PCR of the ileum, and the expression of the tight junction protein ZO-1 was found to increase in the experimental group (Fig. 9c). We also compared the protein expression levels using immunohistochemistry, and the same conclusion was reached (Fig. 9d). To determine whether *F. prausnitzii* has an independent effect on ZO-1 expression, we performed cell experiments and found that live but not inactivated *F. prausnitzii* could increase ZO-1 expression (Fig. 9e).

## Discussion

There is increasing evidence that the gut microbiota plays a key role in the development of atherosclerosis [8, 29]. However, most related studies are descriptive and clinical, and reports on the prevention of atherosclerosis based on specific gut microbes are rare. Yoshida et al. [29] conducted 16S rRNA sequencing analysis of the gut microbiota of 30 CAD patients and 30 control subjects and confirmed through in vivo experiments that *Bacteroides vulgatus* and *Bacteroides dorei* have antiatherosclerotic effects. In comparison to prior studies, this research has advanced the understanding of CAD by categorizing it based on various severity levels and substantially increasing the sample size. By utilizing refined metagenomics and metabolomics techniques for analysing the gut microbiota, this study identified the potential antiatherosclerotic properties of *F. prausnitzii*. Following a series of experiments, it was confirmed that the oral administration of *F. prausnitzii* via gavage protected mice from atherosclerosis by notably reducing their plasma LPS concentration, representing a pioneering discovery in this domain. For this reason, our study showed that oral gavage of *F. prausnitzii* led to a substantial decrease in LPS levels within the intestinal tract, concomitantly enhancing intestinal permeability. These interconnected effects culminated in a marked reduction in LPS in the systemic circulation, ultimately manifesting as potent antiatherosclerotic actions.

*F. prausnitzii*, a dominant gram-negative bacterium within the Clostridium family of Firmicutes, has been associated with signs of gut microbiota dysregulation in various diseases, notably intestinal inflammatory conditions and gastrointestinal tumours [17, 34]. Although a few studies have reported the role of *F. prausnitzii* in the progression of CVD, most of these studies involve population-based sequencing, and few experimental confirmation and mechanistic exploration studies have been performed. Since *F. prausnitzii* produces butyrate, our initial hypothesis was based on the fact that butyrate exerts an antiatherogenic effect. In this study, the faecal short-chain fatty acids of mice were measured. Compared with those in the control group, the levels of butyrate-based short-chain fatty acids did not increase but rather decreased. Combined with the findings of 16S rRNA sequencing analysis, these findings indicate that the decrease in butyrate may be related to changes in the gut microbiota, such as *Akkermansia muciniphila,* caused by *F. prausnitzii* gavage. Through the use of genetic analysis of LPS synthesis by the gut microbiota of mice and the measurement of faecal LPS levels, we found that the level of LPS in the gut decreased after gavage with *F. prausnitzii*. LPS can bind to LPS-binding proteins and chylomicrons in the gut, enter systemic circulation through lymphatic vessels, enhance the inflammatory process, and affect atherosclerosis [35]. In summary, the substantial negative correlation between *F. prausnitzii* abundance and LPS levels, coupled with the observed modifications in the Toll-like receptor 4 signalling pathway in aortic arteries, strongly suggests that *F. prausnitzii* may significantly contribute to the reduction in LPS levels, thereby potentially exerting antiatherosclerotic effects.

Intestinal barrier function refers to the ability of the intestinal epithelium to separate substances in the intestinal cavity and defend against the invasion of bacteria, endotoxins, and foreign antigens. Structural integrity is the foundation of the intestinal barrier. In addition, the mucus barrier and mechanical barrier play major roles in this process. The mechanical barrier refers to the complete and tightly junctional epithelial structure of the intestinal mucosa; this structure is based on intact intestinal mucosal epithelial cells and the tight junctions between epithelial cells. Tight junctions are dynamic multiprotein complexes mainly composed of occludin, claudin, zonula occludens, etc. In the present study, quantitative RT–PCR was used to measure the mRNA levels of three representative tight junction proteins in the ileum of mice. The expression of the tight junction protein ZO-1 was greater in the experimental group than in the control group, which was further verified at the protein level. This finding is consistent with existing research reports [36]. Early studies on mouse colitis models have shown that the supernatant of *F. prausnitzii* can improve intestinal barrier function, which is closely related to tight junction proteins in mice. Consequently, the supernatant of *F. prausnitzii* was shown to enhance intestinal barrier function by affecting paracellular permeability [37]. Subsequent studies on *F. prausnitzii* and its supernatant were reported, and *F. prausnitzii* was shown to also have a preventive effect on a mouse model of chronic low-grade inflammation, where the mechanism was also attributed to the tight junction effect mediated by certain tight junction proteins [38]. However, existing related research has been based mainly on animal experiments, and intervention with *F. prausnitzii* can lead to significant changes in the structure of the gut microbiota. Due to the complexity of interactions between bacteria, it is not possible to directly confirm that *F. prausnitzii* is responsible for this effect. In addition, decreased levels of LPS in the gut can affect gut barrier function. Through in vitro experiments, we have supplemented the shortcomings of the existing research. We first discovered that the application of live *F. prausnitzii* can reduce LPS stimulation in HCT-116 cells and increase ZO-1 protein expression. Although we have not elucidated the molecular mechanism underlying the regulatory effect of ZO-1, our research provides sufficient evidence confirming that *F. prausnitzii* has an independent effect on the intestinal barrier.

From a clinical perspective, our findings confirm that the gut microbiota and gut metabolites can predict the occurrence and development of CAD well and that they can jointly affect amino acid metabolism and vitamin metabolism. To more accurately reflect the sample information when we included the population, we enrolled participants continuously, which resulted in significant differences between the groups. To explain the source of the difference, we used PERMANOVA to determine the contribution of each index to the gut microbiota. The analysis revealed that different types of CAD had the most significant contribution to the difference in the gut microbiota. Furthermore, our study provides strategies for analysing diseases via multiomics gut microbiota sequencing.

This study has several limitations that warrant careful consideration in its interpretation. First, the analysis of the gut microbiota in CAD patients was conducted at a single centre, potentially introducing bias in population-based sequencing experiments. This limitation indicates that our understanding of the compositional characteristics of the gut microbiota in patients with CAD is incomplete. Second, the use of germ-free animals or antibiotic treatments in animal models was not considered, which could have offered additional insights. Furthermore, certain experimental findings necessitate further validation through the expansion of sample sizes. Third, the identification of potential key bacterial species often emphasizes group differences. Importantly, the most significant differences do not necessarily equate to the most substantial impact. The intricacy of microbial communities suggests that the interplay among multiple species might be more influential on disease mechanisms than the dominance of individual species. Future research could explore the roles of various bacteria, including *R. inulinivorans*, and investigate the effects of dynamic changes and multispecies interactions throughout the disease continuum. Longitudinal studies that monitor the microbiome over time could provide crucial insights into these interactions and their implications for disease onset and progression. Such an approach could enhance our comprehensive understanding of the role of the gut microbiome in health and disease, leading to more holistic insights.

### Supplementary Information

**Additional file 1.** Supplementary materials.

**Additional file 2:** **Figure S1.** Analysis of the Gut Microbiota Following Propensity Matching A: Propensity score matching revealed distinct differences in the abundances of specific gut microbiota constituents among the various groups. Matching took into account factors that influence the microbiota composition, namely, clinical indices, host properties, and the use of particular drugs, including statins, ACEIs or ARBs. B: Heatmap of the correlation between differentially abundant gut microbiota constituents and clinical indicators.

**Additional file 3:** **Figure S2.** Gut microbial alterations in patients with or without coronary artery disease (CAD). The number of enterotypes at the genus level (A) and species level (D) was calculated according to the CH index and Jensen–Shannon-based principal coordinate analysis. The distribution of the control group and CAD patients in each group was clustered according to the genus level (B) and the species level (E). Venn diagram at the genus level (C) and the species level (F).

**Additional file 4: Figure S3.** Tolerance Test of *F. prausnitzii* under Simulated Digestive Tract Conditions. A: Gastric fluid tolerance. B: Intestinal fluid tolerance.

**Additional file 5:** **Figure S4.** Evaluation of liver and kidney function between the intervention group and control group. A: Body weight changes over the course of the experimental period (15 samples per group). B: Comparison of the spleen indices (12 samples per group). C: Comparison of the liver indices (12 samples per group). D: Comparison of serum creatinine levels (12 samples per group). E: Comparison of serum ALT levels (9 to 10 samples per group). F: Comparison of serum AST levels (11 to 12 samples per group). ALT: alanine transaminase, AST: aspartate aminotransferase.

**Additional file 6: Figure S5.** Short-chain fatty acid analysis (8 samples per group). The data are presented as the mean ± standard error of the mean. All *P* values were determined by two independent sample t tests.

**Additional file 7: Figure S6.** Linear correlation between *F. prausnitzii* abundance and LPS concentration in the experimental group. A: Correlation with the faecal LPS concentration. B: Correlation with blood LPS levels.

**Additional file 8:** **Table S1.** PCR primers for detection of each species.

**Additional file 9:** **Table S2.** Statistical results of the Adonis analysis.

**Additional file 10.** The total table of metabolites between different degrees of coronary heart disease and control group. The red background is negative ions; The yellow background is positive ions.

## Data Availability

The data supporting the findings of this study are reasonably available from the corresponding authors. The population metagenomic sequencing data supporting the results of this study have been uploaded to the China National Microbial Oligomer Data Centre with accession numbers HRA003277 (except for the following samples: D17, D30, D35, D42, D99 and D100) and HRA003315.
