# Supplementary material for: Faecalibacterium prausnitzii as a potential Antiatherosclerotic microbe
Source: Cell Commun Signal. 2024 Jan 19;22:54. doi: 10.1186/s12964-023-01464-y (PMC10797727; doi:10.1186/s12964-023-01464-y)
Supplement: Supplementary file 1 — Additional file 1. Supplementary materials. [file 12964_2023_1464_MOESM1_ESM.docx]

**Supplementary materials**

**Inclusion and Exclusion Criteria**

The diagnosis of SCAD was based on the presence of chest pain that did not change in pattern in the preceding 2 months ^1^. UA was defined as a normal measurement of cardiac troponin (cTnI) with at least one of the following criteria: prolonged (>20 min) angina pain at rest, new onset angina (Class II or III according to the Classification of the Canadian Cardiovascular Society), recent destabilization of previously stable angina with at least Canadian Cardiovascular Society Class III angina characteristics (crescendo angina), or postmyocardial infarction angina ^2^. MI was defined as a rise and/or fall of cardiac troponin with at least one value above the 99th percentile upper reference limit and with at least one of the following: (1) ischaemia symptoms; (2) new or presumed new significant ST-segment-T wave changes or new left bundle branch block; (3) development of pathological Q waves in the electrocardiogram; (4) imaging evidence of new viable myocardium loss or new regional wall motion abnormality; or (5) identification of an intracoronary thrombus by angiography or autopsy ^3^.

The exclusion criteria for patients were as follows: 1. patients with asymptomatic coronary artery disease, structural cardiomyopathy, pulmonary heart disease, hyperdynamic heart disease, or gastrointestinal diseases. 2. patients with a history of cardiogenic shock within two weeks; 3. patients with severe liver and kidney dysfunction; 4. patients with a history of gastrointestinal surgery in the previous year, antibiotics for more than 3 days in the previous 3 months, and abnormal stool morphology, such as diarrhoea and dry stool.

**Propensity Score Matching**

Propensity score matching (PSM) was performed using DECISIONLINNC software (https://fast.statsape.com). In brief, the analysis employs the mnps function to estimate propensity scores for each sample, enabling calculation of the average treatment effect (ATE), as well as average weights and sample sizes for each group. The data are partitioned into distinct groups and sorted in descending order based on weight. Subsequently, a corresponding number of samples with maximum weights are selected from each group considering their respective sample sizes. Finally, following PSM analysis, these selected samples are merged to form a dataset.

***F. prausnitzii* Survival in the Digestive Tract Assay**

For analysis of the survival of *F. prausnitzii* in gastric and intestinal environments, 3 mL of the bacterial solution was separately added to 27 mL of artificial gastric juice (pH 3, PH1840, PHYGENE, China) and 27 mL of artificial intestinal fluid (pH 6.8, PH1841, PHYGENE, China). The mixtures were vortexed thoroughly under anaerobic conditions before aliquoting ^4^. The cultures were then incubated separately at 37 °C under anaerobic conditions. At various cultivation time points, the bacterial suspensions were diluted, and 100 μL of each suspension was evenly spread onto LYHBHI agar plates using an "L" rod. The plates were subsequently incubated in an anaerobic chamber at 37 °C for 36-48 hours, after which colonies were counted ^5, 6^.

**DNA extraction, 16S rRNA gene amplification, and pyrosequencing (mouse faecal samples)**

Faecal samples were collected from the mice at 24 weeks of age. DNA extractions from mouse faecal samples and 16S rRNA gene amplification and sequencing using the MiSeq system (Illumina, San Diego, CA) were performed by Shanghai Applied Protein Technology Co., Ltd.

The DNA concentration and purity were monitored on 1% agarose gels. The DNA was diluted to a concentration of 1 ng/μl using sterile water. The primers used were as follows: 16S V3-V4: 341F-806R. The 16S rRNA gene was amplified using specific primers with barcodes. All PCRs were carried out in 30 μL reactions with 15 μL of Phusion® High-Fidelity PCR Master Mix (New England Biolabs), 0.2 μM forward and reverse primers, and approximately 10 ng of template DNA. Thermal cycling consisted of initial denaturation at 98 ℃ for 1 min; 30 cycles of denaturation at 98 ℃ for 10 s, annealing at 50 ℃ for 30 s, and elongation at 72 ℃ for 60 s; and a final extension at 72 ℃ for 5 min. The same volume of 1X loading buffer (containing SYB green) was mixed with the PCR products, after which electrophoresis was performed on a 2% agarose gel for detection. Samples with bright main bands between 400 and 450 bp were chosen for further experiments. The PCR products were mixed in equal ratios. Then, the mixed PCR products were purified with an AxyPrepDNA Gel Extraction Kit (Axygen). Sequencing libraries were generated using an NEBNext ® Ultra™DNA Library Prep Kit for Illumina (NEB, USA) following the manufacturer’s recommendations, and index codes were added. The library quality was assessed on a Qubit 2.0 Fluorometer (Thermo Scientific) and an Agilent Bioanalyzer 2100 system. Finally, the library was sequenced on an Illumina NovaSeq 600 platform, and 250 bp paired-end reads were generated.

**Detection of short-chain fatty acids in faeces (mouse faecal samples)**

Faecal samples were collected from the mice at 24 weeks of age. All analyses were performed by Shanghai Applied Protein Technology Co., Ltd. The experimental process was as follows: faecal samples were carefully thawed on ice. Then, 30 mg of each sample was added to a centrifuge tube, and 0.5% phosphoric acid, ethyl acetate, and 4-methylvaleric acid were sequentially added and homogenized while the supernatants were extracted. An Agilent Model 7890A/5975C gas chromatography‒mass spectrometry system (Agilent, Santa Clara, CA, USA) was used for gas chromatography‒mass spectrometry analysis, and an MSD ChemStation (Santa Clara, CA, USA) was used to process the data to quantify the SCFAs.

**α-Diversity, β-diversity and random forest classifier**

The Shannon index, Simpson index, and Pielou index (α-diversity) were calculated with QIIME (v1.7.0) based on the species profiles ^7, 8^. β-diversity was estimated by Bray‒Curtis dissimilarity index matrices using the ANOSIM method in the vegan R package ^9^. These matrices were also used to assess differences in β diversity via ANOSIM. PCoA was carried out for all dimension reduction analyses using the vegan package in R software. P values were corrected for multiple testing with the Benjamin–Hochberg method. A random forest classifier was used for the random forest package in R. Correlations between enriched species and biochemical indicators were tested with Spearman’s correlation.

**PERMANOVA**

Permutational multivariate analysis of variance (PERMANOVA) was performed using the R programming language ^10, 11^. The clinical indicators of the population were summarized and combined with those of the CAD group for analysis. The 'vegan' package in R, which is specifically designed for ecological and community data analysis, was utilized. These results indicate the significance of the differences among the groups.

**Correlation analysis**

Spearman's correlation coefficients were calculated using the 'psych' package in R to conduct correlation analysis, generate a heatmap, and adjust p values ^12^. The results were subsequently visualized as a heatmap using the 'pheatmap' function, which displayed both correlation coefficients and significance indicators.

**Differential analysis of the gut microbiota**

The microbiome analysis was conducted using the ALDEx2 package in the R programming language ^13^. Data preprocessing, including sample selection and normalization, was performed. Subsequently, a compositional log-ratio transformation was applied to the data, and KW scores were calculated to identify taxonomic features associated with different experimental groups. The results were filtered based on a significance threshold of 0.05 and further refined based on their statistical significance. The relative abundance of these taxonomic features across different groups was visualized using boxplots, with the ggplot2 package used for visualization. Additionally, pairwise group differences were assessed using Wilcoxon tests, with p values adjusted using the Benjamini–Hochberg method. The results were visualized using ggplot2, which displayed taxonomic features with significant differences between groups.

**Metabolite analysis**

For the analysis of metabolite data, a univariate analysis method was used, with fold change analysis (FC analysis) and a t test being used. The significance of metabolite changes between two samples was visually displayed by this approach, facilitating the screening of potential marker metabolites. The influence intensity and explanatory ability of the expression pattern of each metabolite on the classification and discrimination of each group of samples were measured using the variable weight value known as variable importance for the projection (VIP), which was obtained through the OPLS-DA model. Biologically significant differentially abundant metabolites were identified through this mining process. Initially, metabolites showing differences between groups were screened out using the criterion VIP>1. Further verification of the significance of these differentially abundant metabolites was conducted using univariate statistical analysis. Metabolites meeting both criteria of multidimensional statistical analysis (VIP > 1) and univariate statistical analysis (P value < 0.05) were considered to be metabolites with significant differences. Metabolite enrichment analysis was performed using the KEGG database.

**Combined multiomics analysis**

The statistically significant species in each group were evaluated via linear discriminant analysis (LDA) of effect size (LEfSe), which employs the nonparametric factorial Kruskal‒Wallis test, Wilcoxon rank sum test and LDA to identify differentially abundant biomarkers between two metadata classes. Differentially enriched KEGG modules of four selected pathways (beta-alanine metabolism, lysine degradation, riboflavin metabolism and valine, leucine and

isoleucine biosynthesis) in metabolism were identified according to their reporter score, calculated from the Z scores of individual KO groups ^14^. A module with a reporter score Z >1.5 (> 90% confidence according to a normal distribution) was considered to indicate significant dysbiosis.

**REFERENCES**

1. Task Force Members, Montalescot G, Sechtem U, et al. 2013 ESC guidelines on the management of stable coronary artery disease: the Task Force on the management of stable coronary artery disease of the European Society of Cardiology . Eur Heart J. 2013;34(38):2949-3003.
2. Petrosyan H, Hayrapetyan H, Torozyan S, et al. Total Ischemic Time on In-Hospital Complication Predictor in ST-Elevation Myocardial Infarction (STEMI) Patients With Renal Dysfunction. Cureus. 2023;15(1):e33903.
3. Thygesen K, Alpert JS, Jaffe AS, et al. Third universal definition of myocardial infarction. Glob Heart. 2012;7(4):275-295.
4. McConnell EL, Basit AW, Murdan S. Measurements of rat and mouse gastrointestinal pH, fluid and lymphoid tissue, and implications for in-vivo experiments. J Pharm Pharmacol. 2008;60(1):63-70.
5. Uriot O, Galia W, Awussi AA, et al. Use of the dynamic gastro-intestinal model TIM to explore the survival of the yogurt bacterium Streptococcus thermophilus and the metabolic activities induced in the simulated human gut. Food Microbiol. 2016;53(Pt A):18-29.
6. Chen D, Chen C, Guo C, et al. The regulation of simulated artificial oro-gastrointestinal transit stress on the adhesion of Lactobacillus plantarum S7. Microb Cell Fact. 2023;22(1):170.
7. Li H, Xu H, Li Y, et al. Alterations of gut microbiota contribute to the progression of unruptured intracranial aneurysms. Nat Commun. 2020;11(1):3218.
8. Yang HT, Xiu WJ, Liu JK, et al. Gut Microbiota Characterization in Patients with Asymptomatic Hyperuricemia: probiotics increased. Bioengineered. 2021;12(1):7263-7275.
9. Yang HT, Xiu WJ, Liu JK, et al. Characteristics of the Intestinal Microorganisms in Middle-Aged and Elderly Patients: Effects of Smoking. ACS Omega. 2022;7(2):1628-1638.
10. Wang X, Yang S, Li S, et al. Aberrant gut microbiota alters host metabolome and impacts renal failure in humans and rodents. Gut. 2020;69(12):2131-2142.
11. Chen J, Zhang X. D-MANOVA: fast distance-based multivariate analysis of variance for large-scale microbiome association studies. Bioinformatics. 2021;38(1):286-288.
12. Yang HT, Liu JK, Xiu WJ, et al. Gut Microbiome-Based Diagnostic Model to Predict Diabetes Mellitus. Bioengineered. 2021;12(2):12521-12534.
13. Fernandes AD, Reid JN, Macklaim JM, et al. Unifying the analysis of high-throughput sequencing datasets: characterizing RNA-seq, 16S rRNA gene sequencing and selective growth experiments by compositional data analysis. Microbiome. 2014;2:15.
14. Wu IW, Gao SS, Chou HC, et al. Integrative metagenomic and metabolomic analyses reveal severity-specific signatures of gut microbiota in chronic kidney disease. Theranostics. 2020;10(12):5398-5411.
