# Supplementary figures and images for: Faecalibacterium prausnitzii as a potential Antiatherosclerotic microbe
Source: Cell Commun Signal. 2024 Jan 19;22:54. doi: 10.1186/s12964-023-01464-y (PMC10797727; doi:10.1186/s12964-023-01464-y)

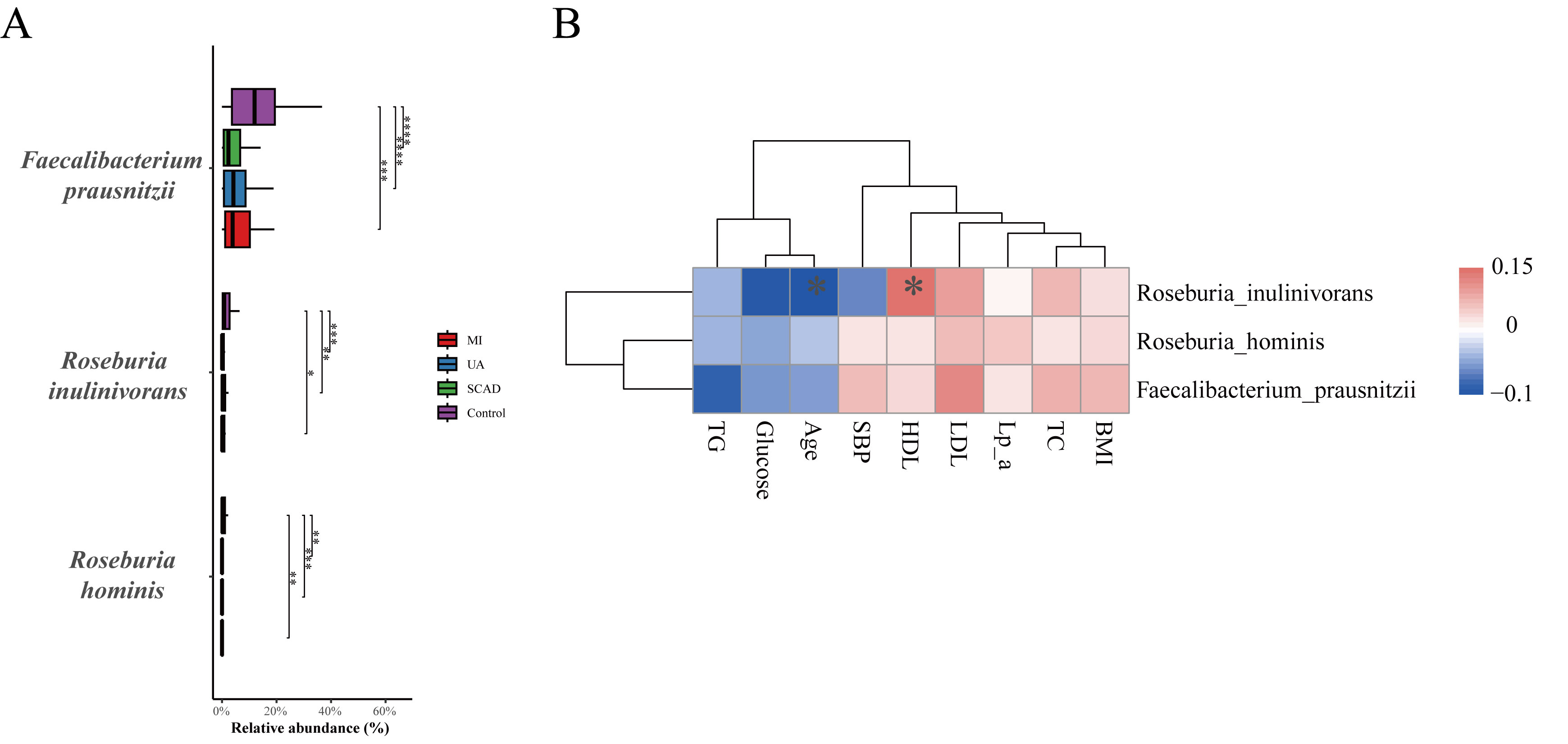

Supplement: Supplementary file 2 — Additional file 2: Figure S1. Analysis of the Gut Microbiota Following Propensity Matching A: Propensity score matching revealed distinct differences in the abundances of specific gut microbiota constituents among the various groups. Matching took into account factors that influence the microbiota composition, namely, clinical indices, host properties, and the use of particular drugs, including statins, ACEIs or ARBs. B: Heatmap of the correlation between differentially abundant gut microbiota constituents and clinical indicators. [file 12964_2023_1464_MOESM2_ESM.jpg]

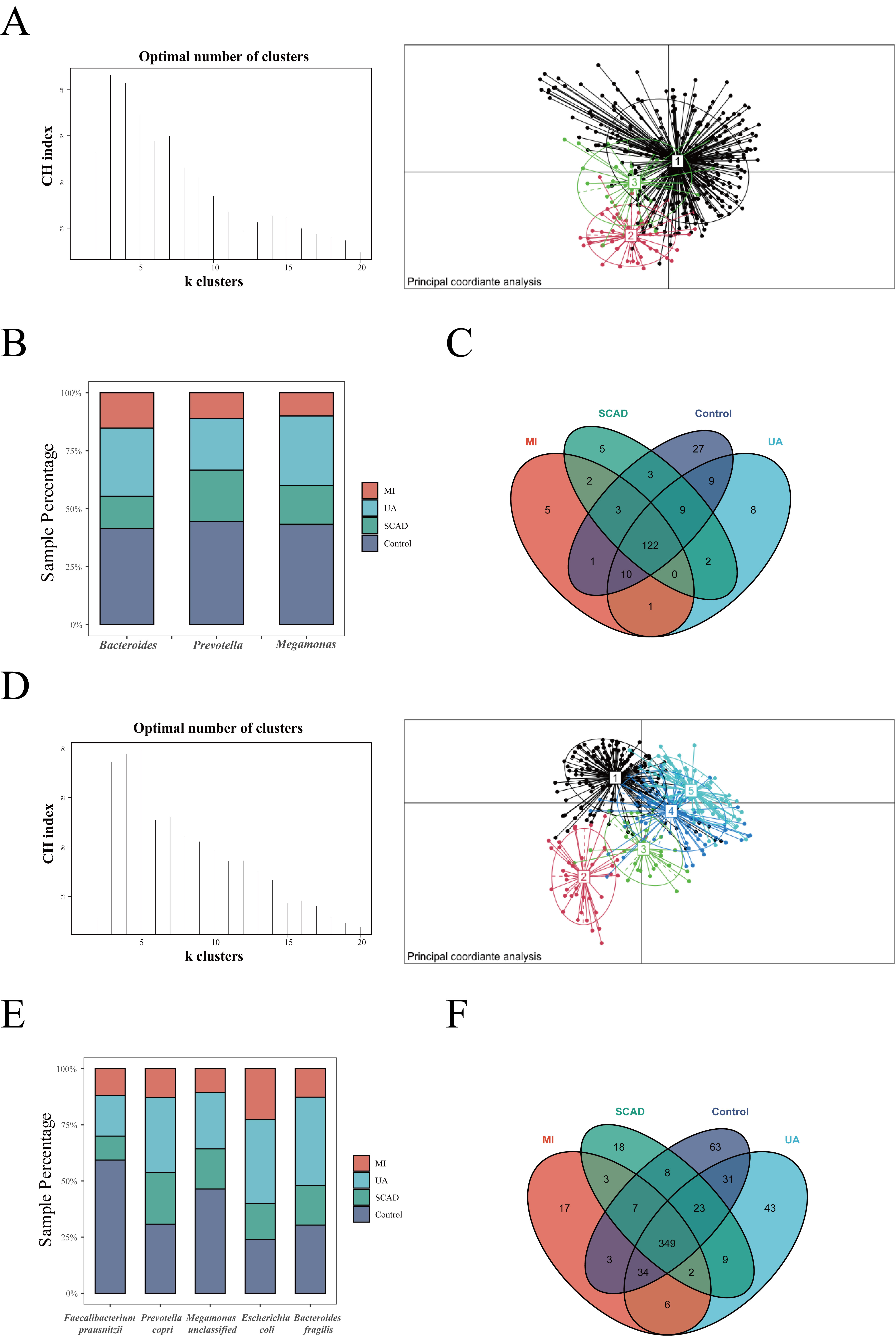

Supplement: Supplementary file 3 — Additional file 3: Figure S2. Gut microbial alterations in patients with or without coronary artery disease (CAD). The number of enterotypes at the genus level (A) and species level (D) was calculated according to the CH index and Jensen–Shannon-based principal coordinate analysis. The distribution of the control group and CAD patients in each group was clustered according to the genus level (B) and the species level (E). Venn diagram at the genus level (C) and the species level (F). [file 12964_2023_1464_MOESM3_ESM.jpg]

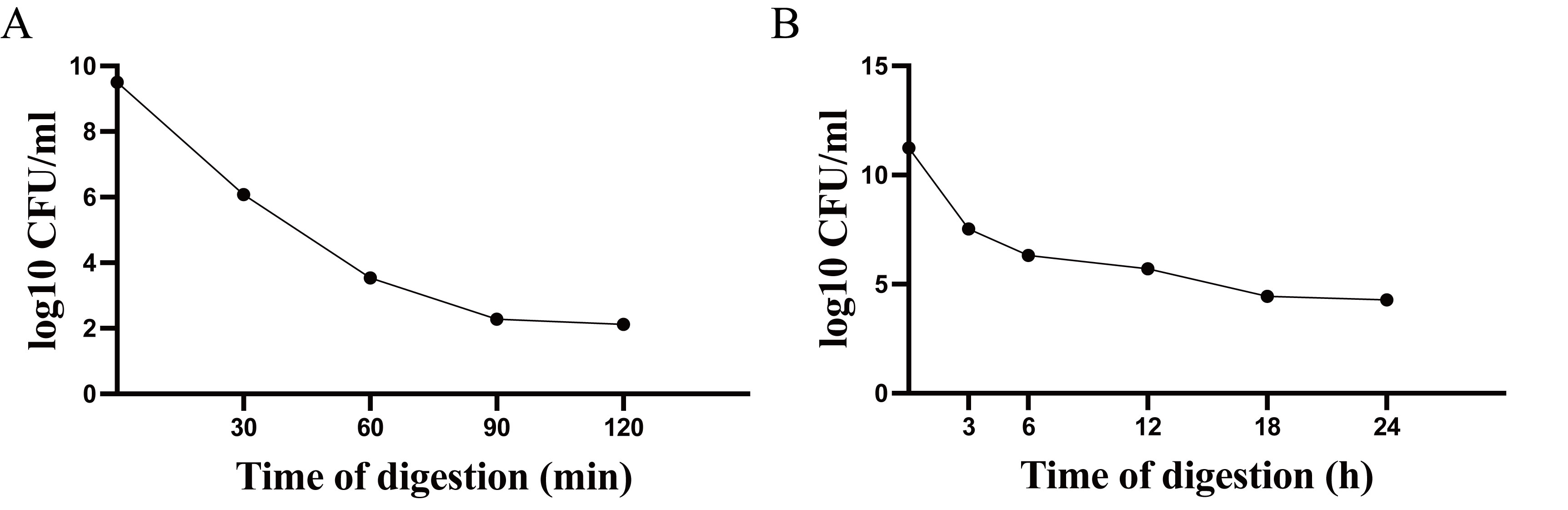

Supplement: Supplementary file 4 — Additional file 4: Figure S3. Tolerance Test of F. prausnitzii under Simulated Digestive Tract Conditions. A: Gastric fluid tolerance. B: Intestinal fluid tolerance. [file 12964_2023_1464_MOESM4_ESM.jpg]

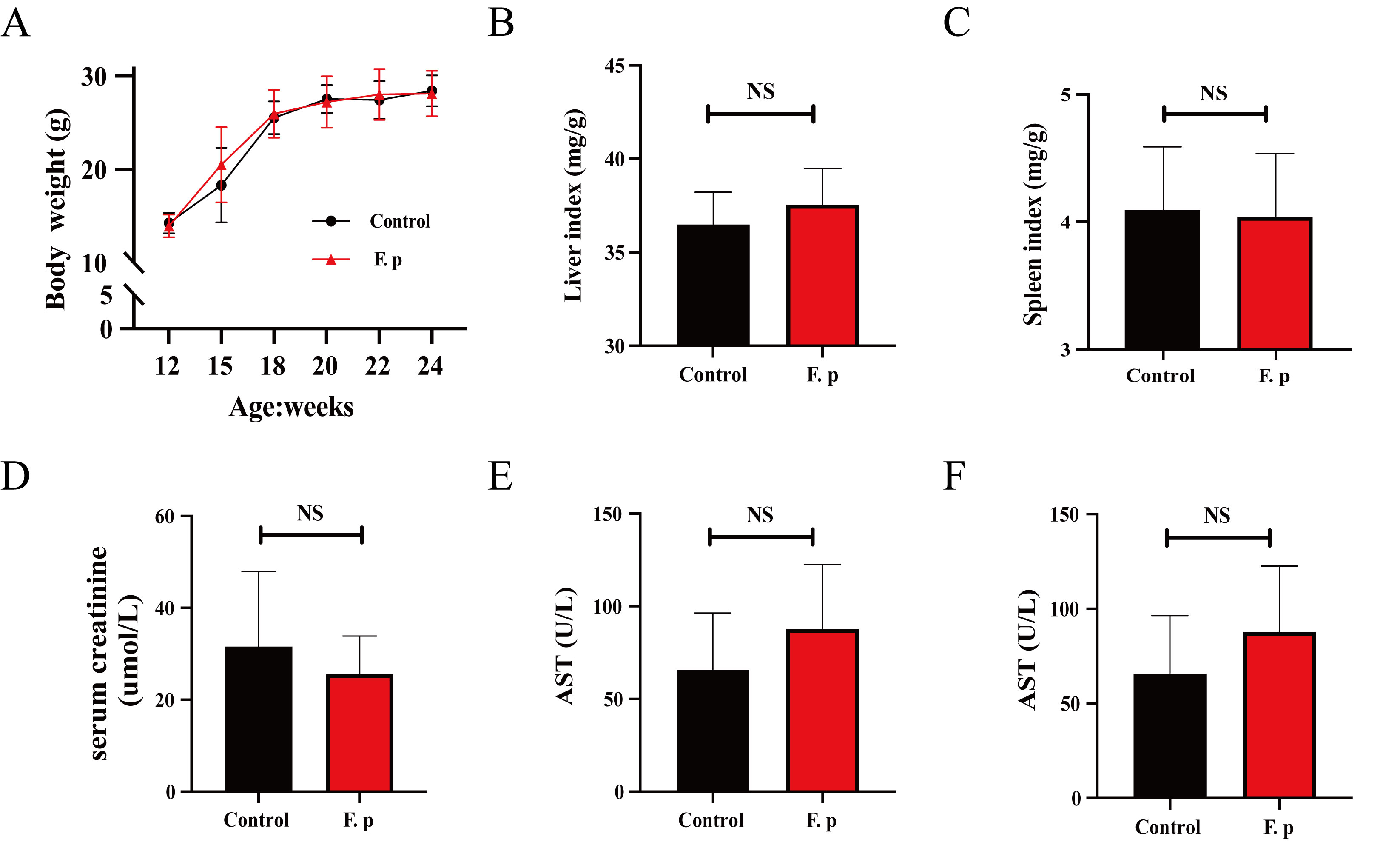

Supplement: Supplementary file 5 — Additional file 5: Figure S4. Evaluation of liver and kidney function between the intervention group and control group. A: Body weight changes over the course of the experimental period (15 samples per group). B: Comparison of the spleen indices (12 samples per group). C: Comparison of the liver indices (12 samples per group). D: Comparison of serum creatinine levels (12 samples per group). E: Comparison of serum ALT levels (9 to 10 samples per group). F: Comparison of serum AST levels (11 to 12 samples per group). ALT: alanine transaminase, AST: aspartate aminotransferase. [file 12964_2023_1464_MOESM5_ESM.jpg]

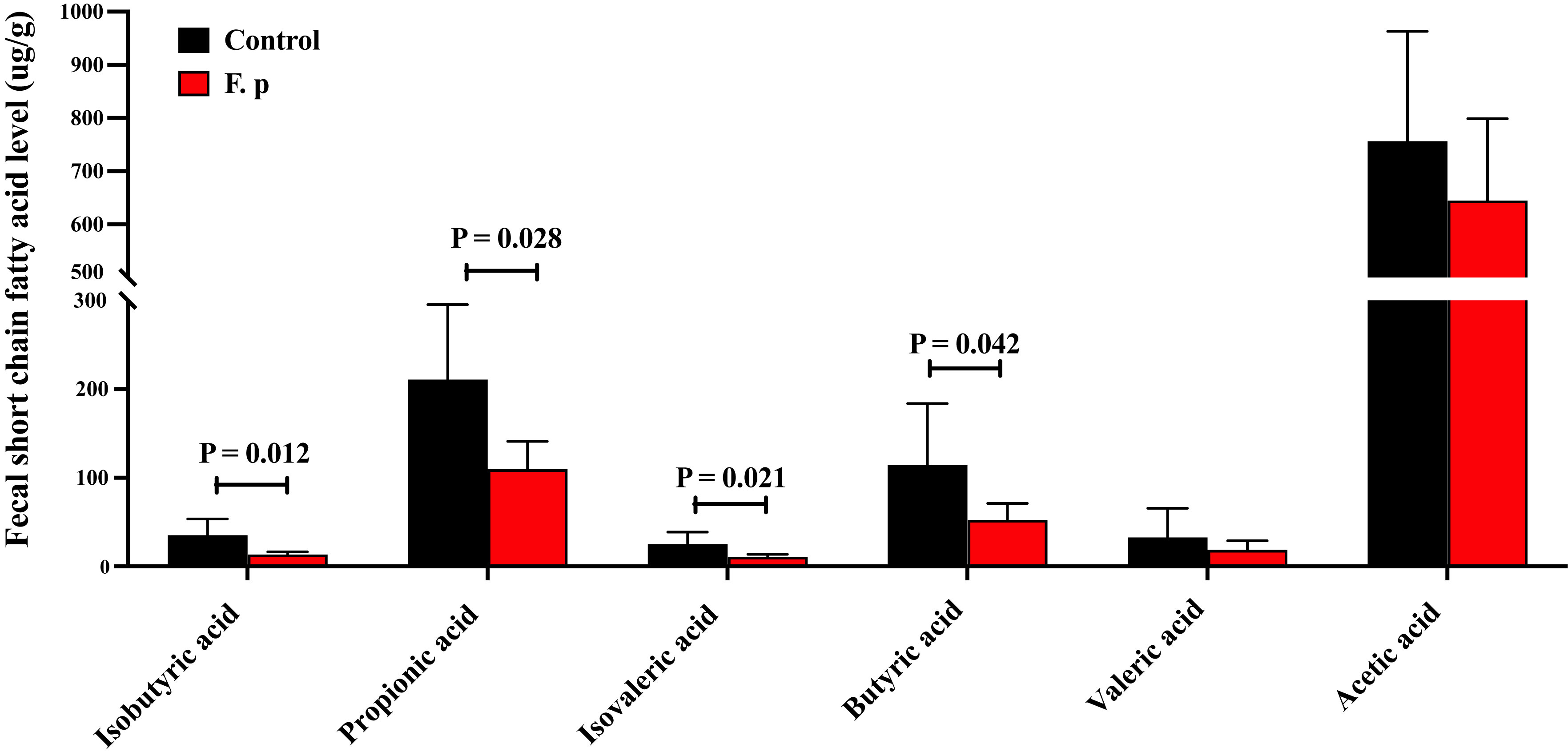

Supplement: Supplementary file 6 — Additional file 6: Figure S5. Short-chain fatty acid analysis (8 samples per group). The data are presented as the mean ± standard error of the mean. All P values were determined by two independent sample t tests. [file 12964_2023_1464_MOESM6_ESM.jpg]

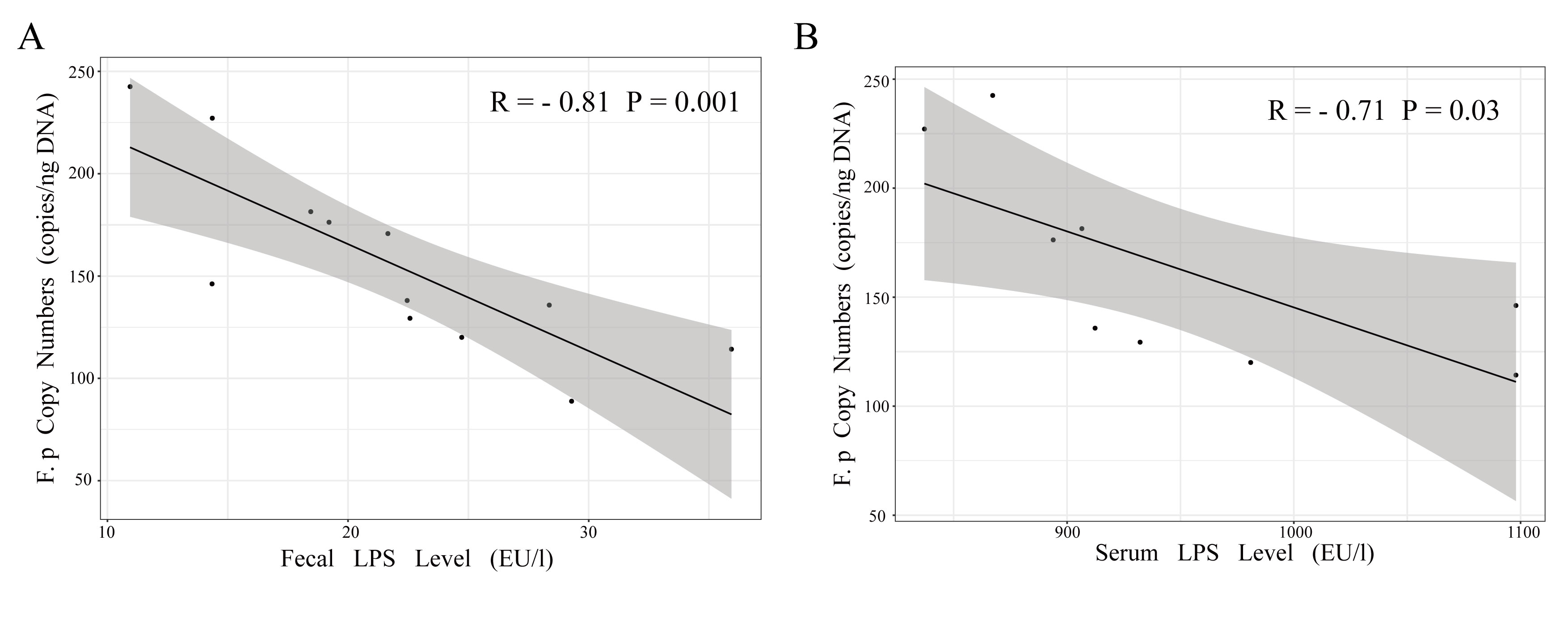

Supplement: Supplementary file 7 — Additional file 7: Figure S6. Linear correlation between F. prausnitzii abundance and LPS concentration in the experimental group. A: Correlation with the faecal LPS concentration. B: Correlation with blood LPS levels. [file 12964_2023_1464_MOESM7_ESM.jpg]
