# Supplementary material for: Faecalibacterium prausnitzii as a potential Antiatherosclerotic microbe
Source: Cell Commun Signal. 2024 Jan 19;22:54. doi: 10.1186/s12964-023-01464-y (PMC10797727; doi:10.1186/s12964-023-01464-y)
Supplement: Supplementary file 8 — Additional file 8: Table S1. PCR primers for detection of each species. [file 12964_2023_1464_MOESM8_ESM.docx]

**Supplementary Table 1. PCR primers for detection of each species**

| **Target species** | **Primer sequence (5′ - 3′)** |
| --- | --- |
| **F. prausnitzii** | forward:GATGGCCTCGCGTCCGATTAG |
|  | reverse:CCGAAGACCTTCTTCCTCC |
| **F4/80** | forward: CTT TGG CTA TGG GCT TCC AGT C |
|  | reverse: GCA AGG AGG ACA GAG TTT ATC GTG |
| **ICAM-1** | forward: CAA TTC ACA CTG AAT GCC AGC TC |
|  | reverse: CAA GCA GTC CGT CTC GTC CA |
| **MCP-1** | forward: CCACTCACCTGCTGCTACTCA |
|  | reverse:TGGTGATCCTCTTGTAGCTCTCC |
| **VCAM-1** | forward: TGC CGG CAT ATA CGA GTG TGA |
|  | reverse: CCC GAT GGC AGG TAT TAC CAA G |
| **CD4** | forward: TCA CAC ATG AAG CAT GTC AGG |
|  | reverse: GCA CTG GTT AGA ATG TGA GTC TG |
| **CD80** | forward: AGT TTC CAT GTC CAA GGC TCA TTC |
|  | reverse: TTG TAA CGG CAA GGC AGC AAT A |
| **CD86** | forward: TGG CAT ATG ACC GTT GTG TGT G |
|  | reverse: ACG TTT GAG CAG ATG GAA ACT CTT G |
| **Foxp3** | forward: CTC ATG ATA GTG CCT GTG TCC TCA A |
|  | reverse: AGG GCC AGC ATA GGT GCA AG |
| **β-Actin** | forward: TGAGCTGCGTTTTACACCCT |
|  | reverse: GCCTTCACCGTTCCAGTTTT |
| **ZO-1** | forward: TTT TTG ACA GGG GGA GTG G |
|  | reverse: TGC TGC AGA GGT CAA AGT TCA AG |
| **Occludin** | forward: ATG TCC GGC CGA TGC TCT C |
|  | reverse: TTT GGC TGC TCT TGG GTC TGT AT |
| **Claudin1** | forward: TCT ACG AGG GAC TGT GGA TG |
|  | reverse: TCA GAT TCA GCA AGG AGT CG |
| **IKK.a** | forward: GGC TGG ACA GCG TCTC TTTA |
|  | reverse: GGT GGA AGA TGG AGCC AGAC |
| **IKK.b** | forward: GTGC CTG TGAC AGC TTA CCT |
|  | reverse: ACTG CGT TTGC ACT TTT GCT |
| **NF-kb** | forward: AAA ATT CGA GTG ACA AGC CTG TA |
|  | reverse: CCC TTG AAG AGA ACC TGG GAG TA |
| **TLR4** | forward: GGG CCT AAA CCC AGT CTG TTT G |
|  | reverse: GCC CGG TAA GGT CCA TGC TA |
| **MAPK** | forward: GCT GAAG CGCC ATTC AAGTT |
|  | reverse: CCT CTGAG CCCT TGTC CAAT |
| **MyD88** | forward: GAT GGTA GCGG TTGT CTC TGAT |
|  | reverse: GAT GCTG GGGA ACTT TCT TC |
| **TAK1** | forward: GAA GGTG GATC CCTG CAC AA |
|  | reverse: CAT GCAA ATAT GCCA GGC CC |
| **TNF-a** | forward:AAA ATT CGA GTG ACA AGC CTG TA |
|  | reverse: CCC TTG AAG AGA ACC TGG GAG TA |
