# Supplementary material for: Faecalibacterium prausnitzii as a potential Antiatherosclerotic microbe
Source: Cell Commun Signal. 2024 Jan 19;22:54. doi: 10.1186/s12964-023-01464-y (PMC10797727; doi:10.1186/s12964-023-01464-y)
Supplement: Supplementary file 9 — Additional file 9: Table S2. Statistical results of the Adonis analysis. [file 12964_2023_1464_MOESM9_ESM.docx]

**Supplementary Table 2. Statistical results of the Adonis analysis**

| **Characteristics** | **SumsOfSqs** | **MeanSqs** | 1. **Model** | **R2** | **p_value** | **p.adjust** |
| --- | --- | --- | --- | --- | --- | --- |
| **Host property** |  |  |  |  |  |  |
|  |  |  |  |  |  |  |
| Age | 1.243 | 1.243 | 3.815 | 0.010 | 0.001 | 0.004 |
| Male | 0.867 | 0.867 | 2.652 | 0.007 | 0.002 | 0.007 |
| Body mass index | 0.817 | 0.817 | 2.500 | 0.007 | 0.001 | 0.004 |
| Systolic blood pressure | 0.387 | 0.387 | 1.179 | 0.003 | 0.224 | 0.342 |
| Diastolic blood pressure | 0.459 | 0.459 | 1.401 | 0.004 | 0.064 | 0.144 |
| Smoking status | 0.456 | 0.456 | 1.389 | 0.004 | 0.097 | 0.187 |
| Drinking status | 0.609 | 0.609 | 1.860 | 0.005 | 0.012 | 0.032 |
| CAD subgroups | 2.879 | 0.960 | 2.968 | 0.024 | 0.001 | 0.004 |
| Hypertension | 0.434 | 0.434 | 1.322 | 0.004 | 0.126 | 0.227 |
| Diabetes | 1.467 | 1.467 | 4.509 | 0.012 | 0.001 | 0.004 |
| **Drug** |  |  |  |  |  |  |
|  |  |  |  |  |  |  |
| Aspirin | 0.706 | 0.706 | 2.155 | 0.006 | 0.001 | 0.004 |
| Clopidogrel | 1.296 | 1.296 | 3.979 | 0.011 | 0.001 | 0.004 |
| Stain | 0.603 | 0.603 | 1.840 | 0.005 | 0.009 | 0.027 |
| Calcium Channel Blockers | 0.221 | 0.221 | 0.673 | 0.002 | 0.915 | 0.915 |
| β-block | 0.346 | 0.346 | 1.055 | 0.003 | 0.352 | 0.500 |
| ACEI or ARB | 0.548 | 0.548 | 1.673 | 0.005 | 0.022 | 0.054 |
| **Clinical index** |  |  |  |  |  |  |
|  |  |  |  |  |  |  |
| Blood Urea Nitrogen | 0.456 | 0.456 | 1.389 | 0.004 | 0.079 | 0.164 |
| Creatinine | 0.832 | 0.832 | 2.545 | 0.007 | 0.001 | 0.004 |
| Uric Acid | 0.336 | 0.336 | 1.023 | 0.003 | 0.424 | 0.545 |
| Glucose | 0.287 | 0.287 | 0.873 | 0.002 | 0.607 | 0.730 |
| Triglyceride | 64.126 | 0.329 | 1.005 | 0.531 | 0.414 | 0.545 |
| Total cholesterol | 74.903 | 0.327 | 0.990 | 0.620 | 0.622 | 0.730 |
| High Density Lipoprotein | 35.756 | 0.319 | 0.961 | 0.296 | 0.904 | 0.915 |
| Low Density Lipoprotein | 69.635 | 0.332 | 1.022 | 0.574 | 0.228 | 0.342 |
| Apolipoprotein AI | 36.002 | 0.319 | 0.957 | 0.297 | 0.915 | 0.915 |
| Apolipoprotein B | 35.942 | 0.324 | 0.980 | 0.297 | 0.718 | 0.808 |
| Lipoprotein(a) | 115.342 | 0.330 | 1.065 | 0.952 | 0.184 | 0.311 |
